# Impact of valganciclovir therapy on severe IRIS-Kaposi Sarcoma mortality: An open-label, parallel, randomized controlled trial

**DOI:** 10.1371/journal.pone.0280209

**Published:** 2023-05-17

**Authors:** Patricia Volkow, Leslie Chavez Galan, Lucero Ramon-Luing, Judith Cruz-Velazquez, Patricia Cornejo-Juarez, Isabel Sada-Ovalle, Rogelio Perez-Padilla, Beda Islas-Muñoz, on behalf of the Kaposi Sarcoma Study Group

**Affiliations:** 1 Infectious Disease Department, Instituto Nacional de Cancerología, Mexico City, Mexico; 2 Integrative Inmunology Laboratory, Instituto Nacional de Enfermedades Respiratorias, Mexico City, Mexico; 3 Cytogenetic Laboratory, Instituto Nacional de Cancerología, Mexico City, Mexico; 4 Department of Research on Tobacco and COPD, Instituto Nacional de Enfermedades Respiratorias, Mexico City, Mexico; University of Ottawa, CANADA

## Abstract

**Introduction:**

High HHV-8 viral load (VL) in Kaposi Sarcoma (KS) has been associated with Severe Immune Reconstitution Inflammatory Syndrome (Severe-IRIS-KS), which can occur after initiating cART, and leads to high mortality, particularly in patients with pulmonary involvement. We investigate if valganciclovir (as an anti-HHV-8 agent) initiated before cART reduces the mortality associated with Severe-IRIS-KS and the incidence of Severe-IRIS-KS.

**Methods:**

Open-label parallel-group randomized clinical trial in AIDS cART naïve patients with disseminated KS (DKS) as defined by at least two of the following: pulmonary, lymph-node, or gastrointestinal involvement, lymphedema, or ≥30 skin lesions. In the experimental group (EG), patients received valganciclovir 900 mg BID four weeks before cART and continued until week 48; in the control group (CG), cART was initiated on week 0. Non-severe-IRIS-KS was defined as: an increase in the number of lesions plus a decrease of ≥one log10 HIV-VL, or an increase of ≥50cells/mm3 or ≥2-fold in baseline CD4+cells. Severe-IRIS-KS was defined as abrupt clinical worsening of KS lesions and/or fever after ruling out another infection following cART initiation, and at least three of the following: thrombocytopenia, anemia, hyponatremia, or hypoalbuminemia.

**Results:**

40 patients were randomized and 37 completed the study. In the ITT analysis, at 48 weeks, total mortality was the same in both groups (3/20), severe-IRIS-KS attributable mortality was 0/20 in the EG, compared with 3/20 in the CG (p = 0.09), similar to the per-protocol analysis: 0/18 in the EG, and 3/19 in the control group (p = 0.09). The crude incidence rate of severe-IRIS-KS was four patients developed a total of 12 episodes of Severe-IRIS-KS in the CG and two patients developed one episode each in the EG. Mortality in patients with pulmonary KS was nil in the EG (0/5) compared with 3/4 in the CG (P = 0.048). No difference was found between groups in the number of non-S-IRIS-KS events. Among survivors at week 48, 82% achieved >80% remission.

**Conclusions:**

Although mortality attributable to KS was lower in the EG the difference was not statistically significant.

## Introduction

Kaposi Sarcoma (KS) and *Pneumocystis jirovecci* pneumonia heralded the beginning of the HIV/AIDS pandemic [1,2]. Before the era of combined antiretroviral therapy (cART), over 30% of AIDS patients developed KS [3,4] which was considered a second epidemic among men that had sex with men (MSM) [5,6]. The incidence of KS dramatically decreased with the advent of cART, though KS-attributable mortality is still high in the first months after cART initiation compared to AIDS patients without KS [7–10]. The high mortality observed shortly after cART initiation is mainly attributable to Severe Immune Recovery Inflammatory Syndrome associated with KS (Severe-IRIS-KS), which presents as an abrupt clinical worsening of KS alongside severe thrombocytopenia and other laboratory abnormalities [11–13] The clinical presentation of Severe-IRIS-KS shares similarities with Kaposi Sarcoma inflammatory Syndrome (KICS) but it has an abrupt onset, can have recurring episodes, and can respond to vincristine/bleomycin administration. Mortality from Severe-IRIS-KS may be as high as 25–40%, [10,11] which highlights the urgency to identify therapeutic interventions to prevent or improve patients with Severe-IRIS-KS.

High HHV-8 VL levels are associated with disease severity and mortality [14,15] and, are considered a risk factor for the development of IRIS-KS [11,15,16]. Thus targeting HHV-8 to diminish VL with drugs such as Ganciclovir and Foscarnet, which have shown *in vitro* and *in vivo* activity against HHV-8 [17–19] could be valuable for preventing Severe-IRIS-KS and for reducing its mortality. Ganciclovir, before the cART era, was used for secondary prophylaxis against Cytomegalovirus (CMV) end-organ disease and was associated with decreased KS incidence [20,21]. A clinical trial showed that valganciclovir reduced oropharyngeal HHV-8 replication [22].

We performed a randomized clinical trial to test the hypothesis that valganciclovir (ganciclovir prodrug, as an anti-HHV-8 agent) started four weeks before cART initiation, in patients with disseminated Kaposi sarcoma (DKS), would reduce the mortality associated to Severe-IRIS-KS and the incidence of Severe-IRIS-KS events. We also evaluated whether or not in the presence of coinfections serum cytokine levels (IL6, IL10, TNF, and IFN-ɤ,) and C-reactive Protein (CRP) correlated with Severe-IRIS-KS occurrence.

## Patients and methods

The protocol was approved by the Comité de Ética en Investigación (Institutional Research Ethics Committee) (015/031/INI) (CEI/950/15) under the Declaration of Helsinki and US Federal Policy for the Protection of Human Subjects and registered at NIH Clinical Trails ID NCT03296553. All participants signed written informed consent.

The protocol was approved by the institutional IRB on July 2015, and we recruited the first patient on October 2015. The translated protocol was uploaded to Clinical Trials on March 2016, and learned one year later than submission to Clinical Trials was incomplete, so the official date for registration to clinical trials was September 2017.

We conducted an open-label parallel-group randomized clinical trial of patients with DKS (all within the context of T_1_I_1_S_1_ Poor Prognosis according to the European Consensus-Based Interdisciplinary Guideline [23] The report of the trial follows the CONSORT guidelines [24].

### Setting

The Instituto Nacional de Cancerología is a referral center for adult patients with cancer located in Mexico City; it has been an Aids Cancer Clinic since 1990. Patients were recruited from October 21, 2015, to September 4, 2018. The last follow-up visit was on August 27, 2019.

Candidates had an initial thorough clinical evaluation including a work-up to diagnose coinfections and rule out other neoplasms that comprised: ophthalmologic evaluation, computed tomography (CT) scan (neck, thorax, and abdomen), bone marrow culture with bone biopsy, Hepatitis B Virus (HBV) and Hepatitis C Virus (HCV) serology, venereal disease research laboratory (VDRL) test and if indicated, a lumbar puncture to rule-out neurosyphilis, Histoplasma urinary antigen and serology, GenXpert MTB/RIF test **and** upper gastrointestinal tract (GIT) endoscopy with biopsy of lesions. Colonoscopy was performed only on patients with diarrhea or lower-GIT bleeding. Biopsies of enlarged lymph nodes were processed for culture and histopathological analysis. If indicated, we performed bronchoscopy with bronchoalveolar lavage (BAL), thoracic Gallium Scan, or PET-FDG scan.

#### Inclusion criteria

Patients >18 years old, HIV+ naïve to cART with DKS, able and willing to provide written informed consent. DKS was defined as the presence of KS pulmonary disease and/or ≥30 KS skin lesions, with or without lymphedema, and/or lymph node involvement, and/or GIT KS involvement (biopsy proven at least in one site).

#### Exclusion criteria

Another concomitant malignancy, Multicentric Castleman Disease (MCD), steroid treatment two months before screening, active HBV or HCV or CMV end-organ disease, or severely ill patients with APACHE II score >15.

*Randomization*. Patients were randomized by blocks of ten; the assigned group was written in closed envelopes: either to the Control Group (CG) to start cART immediately according to current Mexican Guidelines [25], or to the Experimental Group (EG) to receive valganciclovir 900 mg twice daily for 48 weeks and initiate cART at week 4 after randomization.

### Procedures

Patients who at the time of diagnosis presented with extensive KS involvement or bulky oropharyngeal lesions were treated with vincristine 2 mg (reduced to 1 mg if albumin was <3 g/dL) plus bleomycin 15 UI before cART, or at follow-up in case of S-IRIS-KS or persistence of extensive KS lesions.

Visits were scheduled at baseline, and weeks 1, 2, 4, 8, 12, 16, 24, and 48. At each visit, an Infectious Diseases specialist performed a clinical evaluation and skin lesions were photographed. Blood samples were obtained for: WBC count, complete blood chemistry, urine analysis, CRP, D-Dimer, HIV VL with Abbott Real-time, HHV-8, CMV, and Epstein-Barr Virus (EBV) VL ELITe MGB KIT by ELITe InGenius Software, CD4+, and CD8+ cells count and percentage, (flow cytometry, Facs Canto II, Becton Dickinson). CD4/CD8 ratio and plasma levels of interleukin 6, 10 (IL-6, IL-10), tumor necrosis factor (TNF), and interferon-gamma (IFN-ɤ) were measured using a sandwich-type immunoassay, ELISA (Biolegend). Serology for syphilis, HBV, and HCV was repeated at weeks 24 and 48. If patients had an exacerbation of KS outside the scheduled visit they were re-evaluated with laboratory tests and image studies.

### Severity criteria

For this trial, non-severe-IRIS-KS was considered in patients with an increase in the number of KS lesions plus a decrease of ≥one log10 of HIV-1 RNA VL or an increase of ≥50 cells/mm^3^ or ≥2-fold from baseline CD4+ cells [26]. Severe-IRIS-KS was defined as an abrupt clinical deterioration after cART initiation alongside the presence of at least two clinical and at least three laboratory criteria:

Clinical criteria: 1) Fever (without identified concomitant infection), 2) increase in the size or number of KS lesions, 3) exacerbation of lymphedema, 4) appearance or increase of otherwise unexplained lung opacities on the chest images with a negative Gallium-Scan and 4) appearance or increase of volume of pleural effusion.

Laboratory criteria: 1) Thrombocytopenia <100,000 platelets/ml; 2) Anemia (decrease of at least 1 g/dl from previous measure with no obvious bleeding), 3) Hyponatremia <135 mEq/L and 4) Hypoalbuminemia <3.5 g/dL. Patients with severe IRIS KS can respond to bleomycin/vincristine therapy. We defined the resolution of a Severe-IRIS-KS: when platelets normalized, Hb increased (not due to a blood transfusion), albumin and sodium improved, and fever resolved.

### Outcomes

The main outcome was KS-attributable mortality at 48 weeks; secondary outcomes were the incidence of severe-IRIS-KS events, that is the number of patients with a least one severe IRIS-KS event. We also describe in an exploratory way the overall mortality, the mortality in patients with Pulmonary KS, and the total number of IRIS-KS events in the EG and the CG as we observed patients with more than one severe IRIS-KS episode.

Improvement of KS lesions and KS remission were assessed by comparing the size and number of KS lesions from baseline to 48-week visit comparing pictures taken at every visit, and lung CT-scan for cases with pulmonary involvement.

### Statistical analysis

The sample size was calculated for a study power of 80% and an alpha of 0.05, with an estimated event rate (Severe-IRIS-KS-associated deaths) in the control group of 40%, and in the treated group of 5%. The sample number estimated in each group was 19, for a total of 38 patients.

Descriptive statistics were performed, including the number of deaths, and the number of participants with Severe-IRIS-KS, in the CG and EG groups. Comparisons between EG and CG were performed as intention-to-treat (ITT), and as per-protocol analysis, for the primary outcome of mortality attributed to Severe-IRIS-KS by a Fisher´s exact test, and also comparing survival curves by Log-rank test. We also compared the EG and the CG groups by a Fisher´s exact test on the incidence of Severe-IRIS-KS, on overall mortality, and mortality in patients with pulmonary KS. During the study, we observed that some individuals had more than one Severe-IRIS-KS episode during the 48 weeks of follow-up, so we also compared the total number of Severe-IRIS-KS episodes in each group.

The impact of treatment and time on IL-6, IL10, HHV-8 viral load, and C reactive protein were evaluated by fitting linear mixed effect models, and the impact of some covariates (baseline CD4+, CD4+/CD8+ cell ratio, HHV-8 and HIV VL, plasmatic IL-6, CRP, and by the presence of concomitant coinfections) on outcomes by fitting multivariate Poisson models. Analyses were performed using Stata Statistical program.

## Results

**Fig 1:**
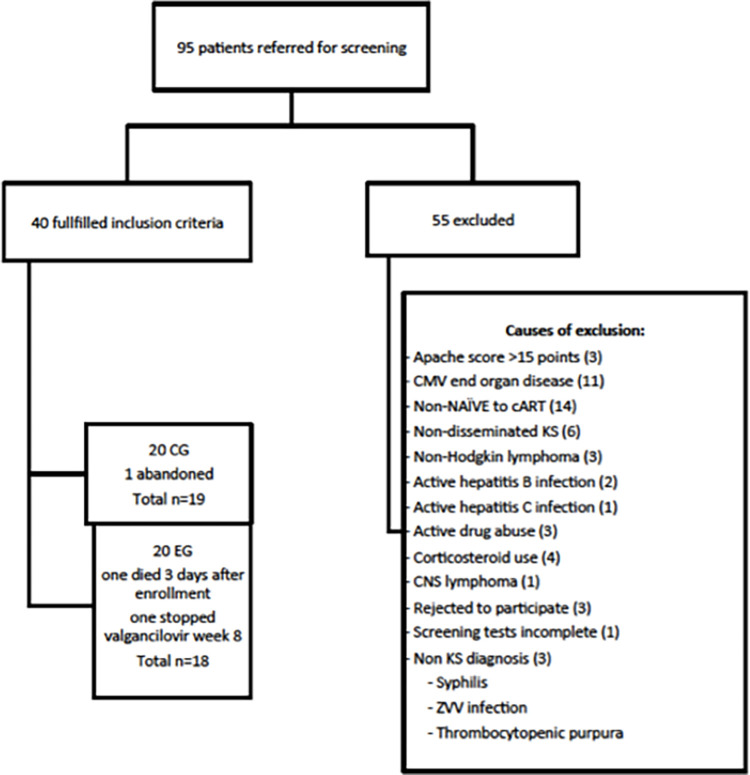
Screening diagram.

A total of 95 male patients were screened, 40 of them were randomized (Fig 1) between October 2015 and September 2018, all of them Hispanic MSM, with a median age of 31 years (IQR 26–36).

The main characteristics of the experimental and control groups are depicted in Table 1.

**Table 1:** Prevalence of coinfections, HIV, Human Herpes virus 8 (HHV-8), Cytomegalovirus (CMV), and Epstein Barr virus (EBV) viremia, laboratory parameters, and cytokines in the experimental and control groups.

	Total N = 40 (%)	Experimental N = 20 (%)	Control N = 20 (%)	P
Median Age years (IQR)	31 (26–36)	30.5 (25.5–35.5)	31(26.5–37)	0.53
*Mycobacterium avium complex*	4 (10)	3 (15)	1 (5)	0.29
Histoplasmosis	7 (17.5)	5 (25)	2 (10)	0.21
Syphilis	10 (25)	4 (20)	6 (30)	0.46
Neurosyphilis	3 (7.5)	2 (10)	1 (5)	1
Parasites	3 (7.5)	1 (5)	2 (10)	0.54
*Helicobacter pylori*	7 (17.5)	4 (20)	3 (15)	0.67
*Pneumocystis jirovecci* pneumonia	2 (5)	1 (5)	1 (5)	1
HHV-8 + CMV+EBV (viremia)	9 (22.5)	3 (15)	6 (30)	NR
HHV-8 + EBV viremia	10 (25)	6 (30)	4 (20)	NR
HHV-8 + CMV viremia	9 (22.5)	6 (30)	3 (15)	NR
Only HHV-8 viremia	7 (17.5)	4 (20)	3 (15)	NR
Only CMV viremia	1 (2.5)	0	1 (5)	NR
Only EBV viremia	2 (5)	1 (5)	1 (5)	NR
Non-EBV, CMV or HHV-8 viremia	2 (5)	0	2 (10)	NR
Any CMV, EBV or HHV-8 viremia	38 (95)	20 (100)	18 (90)	NR
Median CD4+ cells/ml. (IQR)	69 (36–119)	71 (34–110)	62 (38–145)	0.49
Median CD8+ cells/ml. (IQR)	715 (395–1178)	799 (499–1226)	668 (343–119)	0.4
CD4/CD8 ratio (IQR)	0.09 (0.05–0.18)	0.09 (0.05–0.15)	0.1 (0.05–0.22)	0.48
Log10 HHV-8 (IQR)	3.2 (2.58–3.77)	3.46 (3–4.1)	2.9 (2.4–3.7)	0.16
Log10 HIV (IQR)	5.3 (4.6–5.8)	5.3 (4.6–5.6)	5.3 (4.6–5.9)	0.58
Log10 CMV (IQR)	2.4 (1.6–3)	2.4 (1.6–2.9)	2.3 (1.6–3)	0.72
Log10 EBV (IQR)	2.4 (1.9–3.4)	2.4 (1.9–3.6)	2.5 (1.9–3.3)	0.9
IL 6 pg/ml (IQR)	20 (12.1–35.9)	22.8 (15.5–43.9)	14 (10.4–23.7)	0.02
IL10 pg/ml (IQR)	19.7 (11.1–41.5)	20.8 (10.7–37.2)	17.9 (11.8–41)	0.91
TNF pg/ml (IQR)	16.81 (5.2–31.1)	18.4 (4.39–25.9)	14.3 (4.39–25.9)	0.27
IFN pg/ml (IQR)	10.4 (6.4–24.5)	9 (5.71–20)	13.2 (3.9–25.9)	0.63
C reactive protein (IQR)	1.3 (0.68–2.8)	1.16 (0.73–2.55)	1.9 (0.64–3)	0.53
Hemoglobin (g/dL) (IQR)	11.75 (10–13.5)	11.85 (10–13.75)	11.7 (10–13)	0.57
Platelets (cells/ml) (IQR)	176,500 (139,000–262,000	183,500 (126,500–2,925,003)	172,000 (141,500–225,000)	0.43
Albumin (g/dl) (IQR)	3.6 (3–3.9)	3.6 (3–3.8)	3.5 (3–4)	0.53
Sodium Sodium (IQR)	13 (134–140)	137 (133–140)	136 (134–140)	0.29

Reference normal values: IL6 3.4 pg./ml, IL10 9.1 pg./ml, TNF 8.1 (for IFNγ we use the average measurement in samples of 30 healthy subjects 1.8 pg/ml SD 0.8pg/ml).

Thirty-five (87%) patients had HHV-8 viremia at baseline alone or in combination with EBV or CMV (19 EG and 16 CG). Nine (22.5%) patients had pulmonary KS involvement four (20%) in the CG group and five (25%) in the EG. Eleven patients (27.5%) were diagnosed with HIV-polymorphic lymphoproliferative disorder [27]. The prevalence of syphilis, mycosis, mycobacterial and parasitic infections as well as laboratory parameters and cytokines levels did not differ between groups, except for IL-6, which was significantly higher in EG (Table 1).

### ITT analysis

In the ITT analysis comparing all forty randomized patients, the overall mortality was the same, 3 deaths in each group, and the proportion of deaths attributed to Severe-IRIS-KS did not differ significantly between groups, none in the EG and 3 in the CG (p = 0.11 Fisher´s exact test, P = 0.09 Log-rank test)(see Table 2). In the CG group, all deaths were attributable to Severe-IRIS-KS, occurring 70, 88, and 99 days after randomization. In the EG group, one death due to septic shock occurred three days after enrollment, one 89 days post-randomization from an opioid overdose, and one 325 days post-randomization due to H_1_N_1_ influenza pneumonia (Table 2).

**Table 2:** Outcomes of the trial: Mortality and severe-IRIS-KS events.

	Experimental n = 18	Control n = 19	P (Fisher exact test unless otherwise specified)
Primary outcome			
KS-attributable deaths at 48 weeks (ITT analysis)	0/20	3/20	p = 0.11 p = 0.09 Log-rank test**
Total number of deaths at 48 weeks	3/20	3/20	p = 1.0 p = 0.75 Log-rank test.
Secondary outcomes			
Participants with at least one Severe-IRIS-KS event	2/18	4/19	P = 0.66
Total number of Severe-IRIS-KS episodes in the group	2	12	P = 0.24
The incidence rate of Severe-IRIS-KS events in the group (per 100 patient-observation days)	0.038	0.21	IRR 0.20 (0.04–0.9)

**similar results in per protocol analysis with 18 patients in the EG and 19 in the CG.

*Incidence rate was the number of IRIS-KS events (could be more than one per patient in the control group) divided by the total days of observation, 5255 in the control group and 5657 in the experimental group.

In the per-protocol analysis, we included 37 patients, 19 in the CG (one patient abandoned the protocol on week 12) and 18 in the EG (one patient died three days after randomization and another stopped valganciclovir on week 8.

Valganciclovir did not reduce significantly the number of patients with at least one Severe-IRIS-KS episode, 2/18 in the EG and 4/19 in the CG (P = 0.66), Fisher´s exact test) as depicted in Table 2.

Four patients developed 12 episodes of Severe-IRIS-KS in the CG Incidence Rate (IR) (0.21 per 100 patient-observation days) and two patients developed one episode each in the EG (IR 0.038 per 100 patient-days, IRR 0.20, 95%CI 0.04–0.9, *p* = 0.04).

In a multivariate Poisson model, a higher HHV8 Log10 viral load (IRR 1.5, 95%CI 1.2–2.0, p = 0.002), IL6 (IRR 1.07, 95%CI, 1.02–1.12, p = 0.004) and CRP at baseline (IRR 1.1, 95%CI 0.96–1.27) increased the risk to develop Severe-IRIS-KS and valganciclovir use significantly decreased the risk of Severe-IRIS-KS occurrence (adjusted IRR = 0.05, 95%CI 0.003–0.6 p = 0.02) (Table 2).

We performed a linear mixed effect model analysis for longitudinal data for 4 variables IL-6, IL10, HHV-8 viral load, and CRP; the impact of treatment was not significant, at the p<0.05 level and with time, only CRP decreased significantly (P = 0.034).

Of the nine patients with pulmonary KS, three of the four in the CG group died of Severe-IRIS-KS, whereas none of the five patients in the EG died (Fisher´s exact test, p = 0.048). No difference was found in the occurrence of non-severe-IRIS-KS events: 15 in the EG (from 7 patients) and 17 in the CG (from 8 patients) and eight patients in each group did not develop any IRIS event.

There were no differences in the number of patients receiving or the number of cycles of vincristine/bleomycin administered. Seven patients in each group did not receive chemotherapy, four in each group had one cycle, eight had two cycles (CG = 3; EG = 5), three patients had three cycles (CG = 2; EG = 1), one patient had four cycles CG and three patients received five cycles (CG = 2; EG = 1).

At week-48 follow-up 33 patients were alive and one CG was lost to follow-up. There were no group differences in remission rates; 12 (36.3%) achieved complete remission (six in each group); 15 (45.4.7%) achieved 80–95% remission (CG = seven, EG = eight); and six (18%) 50–70% remission (three in each group). We did not document any relapses or non-responders.

#### Comparison baseline *vs*. week-48 measurements

In survivors most measurements improved from baseline to follow-up at week 48; the rate of improvement did not differ between groups. The median CD4+ cells count was 69 cells/mL (IQR 36–119) at baseline and 179 cells/mL (IQR 132–516) at week-48. The median CD8+ at baseline was 715 cells/mL (IQR 395–1178) and 639 cells/mL (IQR 639–673) at week-48. The median CD4+/CD8+ ratio was 0.1 (IQR 0.05–0.22) at baseline and 0.34 (0.16–0.48) at week-48.

**Fig 2:**
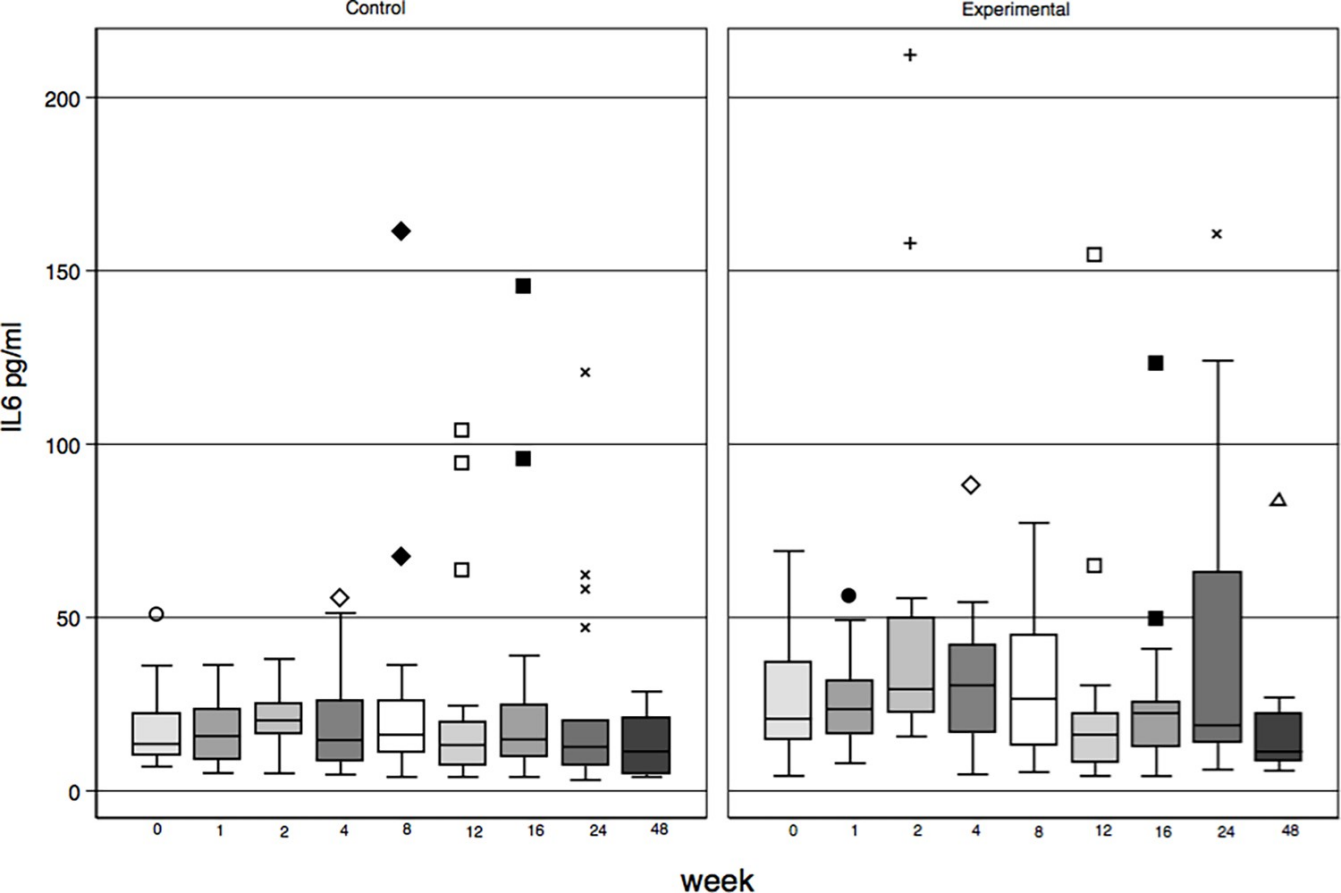
IL6 levels by group by protocol scheduled visit.

At baseline, the median of IL-6 was higher for the EG than for the CG: 22 pg/ml; (IQR 15.5–43.9) vs 14 pg/ml, (IQR10.4–23.7); p = 0.02) (normal <3.4 pg/ml); Fig 2.

Baseline levels for IL-10 19.7 pg/ml, (IQR 11.1–41.5; normal <9.1 pg/ml); IFN-ϒ 10.4 pg./ml, (IQR 6.4–24.5, normal = 1.8pg/ml SD± 0.8pg, RV 4 pg/ml [28] and TNF 19.7 pg/ml, (IQR 11.1–41.5, RV = 8.1 pg/ml) and CRP at baseline was 1.3 (IQR 0.68–2.28) and did not differ between groups (Table 1).

At week 48, IL6 levels for those who survived and were not lost to follow-up (n = 33) were 11.3 pg/ml (IQR 7.4–21), IL10 13.8 (IQR 7.3–36), IFN-ϒ 10.7 pg/ml (IQR 5.6–21.2), and TNF 13.05 pg/ml (IQR 7.7–21.2) there were no differences between groups.

**Fig 3:**
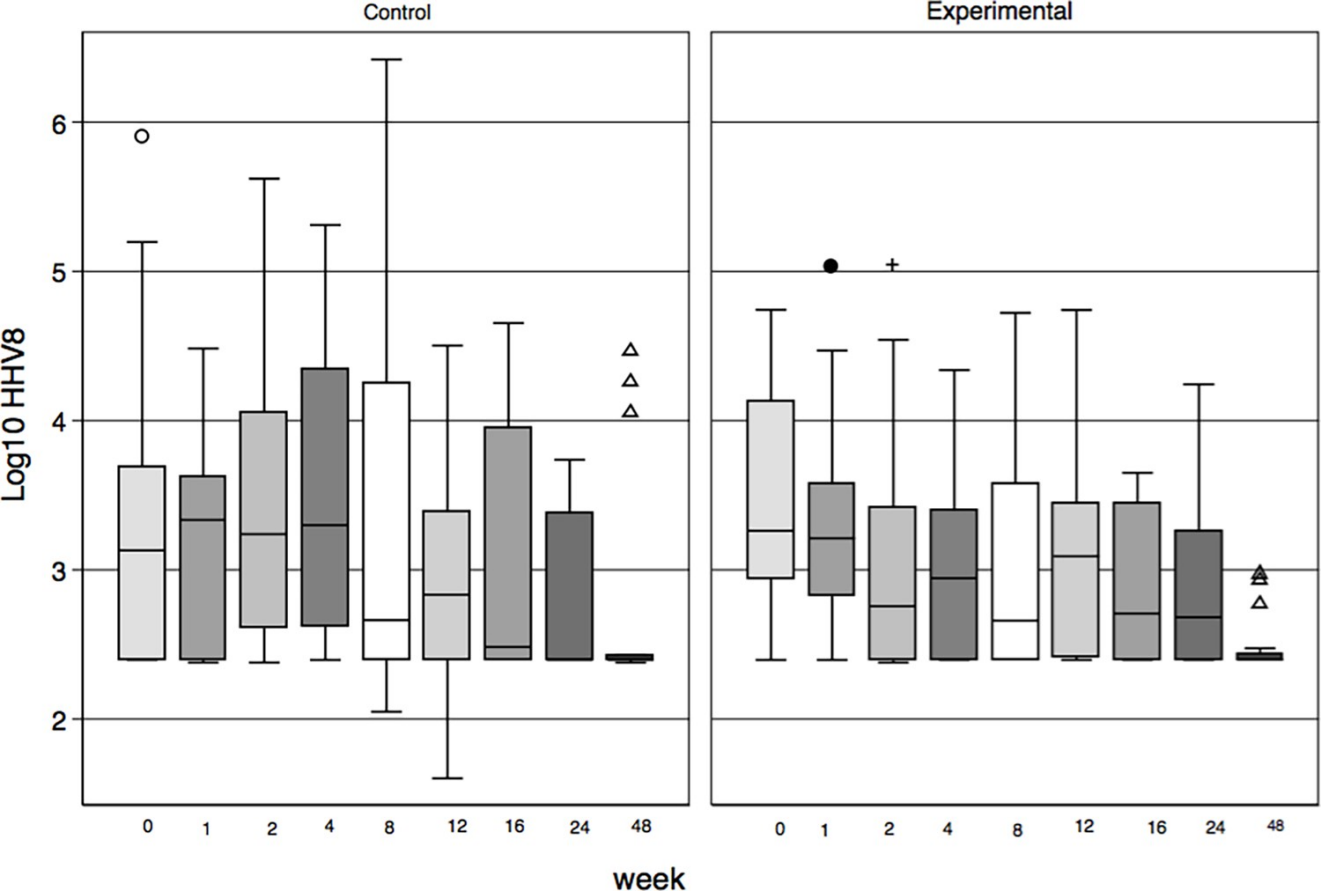
Log10 HHV-8 viral load by group and scheduled visit. The three patients in the control group who died from S-IRIS-KS died within 12 weeks after the start of cART.

At week 48, 23 patients (65%) had undetectable HHV-8 VL, 13 in EG, and ten CG. Three had detectable HHV-8 VL in EG (the highest measurement was 929 copies/ml) and six in CG (three of them with HHV-8 VL >10000 copies/ml) Fig 3. Thirty-one patients (93.9%) had undetectable HIV VL, (<45 copies/ml) and two had HIV viremia (6^.^1%), both with adherence problems.

#### Cytokines according to Severe-IRIS-KS

The median baseline IL-6, IL-10 and CRP levels in the patients who developed Severe-IRIS-KS was significantly higher than in patients without Severe-IRIS-KS events: IL6 36 pg/ml, IQR 16.1–50 vs. 15.5 pg/ml, IQR 11.7–22 (*p* = 0.018) and IL10 76.8 pg/ml, IQR 21–1075 vs. 15.1 pg/ml, IQR 9.9–32, (p = 0.01), CRP 3.4, IQR 3.3–10.1, vs. 1.1, IQR 0.49–1.9, (p = 0.001).

There were no differences in baseline TNF or IFN-ϒ levels among patients according to the development of Severe-IRIS-KS: TNF 16.7. pg/ml IQR 3.4–16.7 vs. 18.07 pg/ml, IQR 6.3–31.1 (p = 0.85), IFN-ϒ 15.9 pg/ml, IQR 8–82.5 vs. 9.3 pg/ml, IQR 5.8–18.7 (p = 0.1).

S1 Fig. depicts the CD4+ cell levels; HHV-8 and HIV viral load for each visit for one patient from the CG and S2 Fig. a participant of EG, both with pulmonary involvement. The Severe-IRIS-KS events are shown along with the outcomes.

S3 Fig. depicts measurements in one patient who stopped valganciclovir on week 8 and resumed it on week 24. The patient developed Severe-IRIS-KS after valganciclovir discontinuation when HHV-8 VL rebounded and CD4+ cells increased, and when he resumed treatment, HHV-8 VL decreased and the Severe-IRIS-KS episode ceased, achieving 90% remission at week-48.

#### Adverse events

Complete adverse events are displayed in Table 3.

**Table 3:** Adverse events.

Adverse Event	Total N = 37	Experimental N = 18	Control N = 19
Severe neutropenia <500–100 cells/mm^3^	5	3*	2
Mild neutropenia ≥500 cells/mm^3^	3	2^+^	1ǂ
Febrile Neutropenia	1	1	0
Cellulitis	6	4	2
Allergic Rash†	8	4	4
*C*. *difficile* infection &	1	1	0
Pneumonia	2	2	0
Multifocal leukoencephalopathy	1	1	0
UTI *E*.*coli* ESBL	2	0	2
Opioid Overdose	1	1	0
Influenza A H1N1	1	1	0
Abdominal sub occlusion	1	1**	0
Oral Mucosa dysplasia	1	0	1
Myositis	1	0	1 §
Acute necrotizing ulcerative gingivitis	1	1	0
Lower limb thrombosis	1	1	0
Heaviness and mild pain in the legs	1	0	1
Suicidal ideation	1	1	0
Neurologic disturbance	2	1	1

* All three patients had disseminated histoplasmosis.

ǂ One patient in each group had *Mycobacterium Avium Complex* (MAC) infection.

† Four patients had rash attributed to efavirenz and four due to TMP/SMX.

& One patient in the experimental group presented *with C*. *difficile* infection with one relapse.

**Attributable to MAC immune reconstitution inflammatory syndrome with abdominal lymph node enlargement.

§ Attributed to Tenofovir.

The use of Filgastrim was significantly greater in EG OR 3.13 *p* = <0.005.

Ten patients acquired a sexually transmitted disease (STD) during the study: eight with syphilis, three at week 24 and five at week 48, one with gonococcal pharyngitis, and one with pediculosis pubis.

## Discussion

In this randomized clinical trial, we evaluated the effect of valganciclovir on DKS patients who were cART naïve, with severe immunosuppression, comorbid coinfections, and variable levels of HHV-8 viremia. We found a non-significant lower KS attributable mortality (*p* = 0.09) in the EG compared to the CG groups, and no reduction on the overall mortality or the number of patients with Severe-IRIS-KS. Exploratory sub-group analysis identified benefits in patients with high baseline HHV-8 viral load, and in those with pulmonary KS, the latter associated with a high risk of death, The sample size was small, due to an overestimation of the impact of valganciclovir, and to unexpected low mortality, probably due to the extensive investigation and treatment of co-infections, not performed before this study. The mortality estimations derived from retrospective studies are likely with underdiagnosis and undertreatment of coinfections and high mortality. KS is an angioproliferative disease caused by HHV-8, mediated by cytokines in severely immunosuppressed individuals [29]. HHV-8 replication correlates with extensive KS and disease progression [15,30,31]. Like all herpes viruses, HHV-8 has a latent and lytic replication phase [32–34] and genetic machinery that mimics human oncogenes [29]. They also promote cell division of infected cells, inhibit apoptosis, modulate inflammation, and induce angiogenesis [35] but not cell immortalization [36]. KS is considered a multicentric, polyclonal disease [4,37,38].

Several inflammatory cytokines promote the lytic phase of the virus, including [39] IL-6, oncostatin, alfa-TNF, platelet-derived growth factor (PDGF), vascular endothelial growth factor (VEGF) [40], and N-kappa B, IFN-ϒ and VEGF from the HIV-Tat protein [41].

HIV-induced -immunodeficiency; a *syne qua none* factor for KS development [36,42,43], and coinfections with opportunistic pathogens lead to a release of inflammatory cytokines and thus lytic activation. We found high levels of the four cytokines studied (IL-6, Il-10, IFNϒ, and TNF) at baseline (Table 1). IL-6, IL-10, and CRP but not IFN-ϒ and TNF were significantly higher among patients that developed Severe-IRIS-KS. High IL-10 levels have been demonstrated in patients with DKS [44].

In this cohort, we found a high prevalence of multiple infections including syphilis (25%), Histoplasmosis and *Helicobacter pylori infection* (17.5% each), MAC in 10%, parasites, and a diverse combination of HHV-8, CMV, and EBV viremia. In the early years of the AIDS epidemic, similar data were published in DKS case reports; and even it was postulated that these coinfections particularly CMV and other STDs could participate in the pathogenesis of DKS [2,45]. Despite evidence of the high prevalence of coinfections in HIV patients [46], only opportunistic infections are addressed in KS guidelines [23,47]. Coinfections in DKS patients should be diagnosed and treated to avoid the vicious cycle of inflammatory cytokines production that promote lytic phase activation and thus HHV-8 replication and in consequence increase in HHV-8 VL [39].

Valganciclovir diminishes HHV-8 VL reducing inflammatory cytokine release when immune reconstitution starts after cART initiation. In S3 Fig, HHV-8 VL increases in parallel to CD4+ cells rising during a Severe-IRIS-KS event in the patient who stopped valganciclovir while continuing to receive cART.

Patients with extended disease or Severe-IRIS-KS were treated with 2 mg vincristine and 15 UI bleomycin. This regime was well tolerated; it is not myelosuppressive and does not require concomitant administration of steroids, a recognized amplifier of KS proliferating signaling [48].

The delay in the initiation of cART while giving an anti-HHV8 agent has the same basis as when cART initiation is delayed in CMV chorioretinitis or cryptococcal meningitis, [49–52] and is to diminish the risk of IRIS development and its deleterious consequences to the patient’s health.

Our definition of Severe-IRIS-KS shares similarities to KICS criteria [53,54], the main difference is the temporal relation to cART initiation, the abrupt initiation of symptoms (fever, increase in SK lesions, lymphedema or pleural effusion, rapid decrease in platelets, Hb, sodium, and albumin) and the response to vincristine/bleomycin [55].

DKS patients typically have high mortality, particularly those with pulmonary involvement [56,57], in our CG three out of four patients with pulmonary KS died of Severe-IRIS-KS *vs*. none of five in the EG group, a significant difference.

We acknowledge some limitations in our study. First, the sample size was small as we overestimated the expected impact of valganciclovir treatment on mortality, and this may explain a mostly negative trial. The studied patients arrived with advanced HIV disease, severely immunosuppressed, an unlikely situation in high-income countries but not in middle and low-income countries where late HIV diagnosis is still an important proportion of newly diagnosed HIV-infected patients. Patients like those described here are likely found in many other middle and low-income countries still nowadays.

However, the trial even if considered a pilot study, leads to several interesting new avenues for this group of severely ill patients, including the utility to perform a thorough investigation of co-infections, measuring the viral load of HHV-8 and utilizing an antiviral agent that inhibits its replication.

From our perspective is important in patients with DKS to address four elements of KS pathogenesis when initiating treatment: (1) Exhaustive search and treatment of co-infections (2) control of HHV-8 viremia with valganciclovir (or another anti-HHV8 active agent) [17,19]. In our hospital, we start valganciclovir in cART naïve DKS patients with an HHV-8 VL of ≥ 1000 copies/ml and/or pulmonary involvement before initiating antiretroviral therapy, and in those with increasing HHV-8 viral load four weeks after starting cART to reduce HHV-8 replication and thus inflammation, proliferation, and differentiation of spindle cells promoted by HHV-8. [58], (3) suppression of HIV viremia with cART to allow immune restoration [59] and (4) suppression of spindle-cell proliferation with antimicrotubule agents that do not exacerbate immunosuppression.

## Conclusions

Valganciclovir reduced the number of episodes of Severe-IRIS-KS and also the attributed mortality although this change was not statistically significant.

## Supporting information

S1 Checklist CONSORT 2010 checklist of information to include when reporting a randomised trial*. (DOC)

S1 Fig Clinical evolution, CD4+ cell count, HIV, and, HHV-8 viral load for each visit in a patient from the control group, with pulmonary involvement diagnosed after cART initiation (unmasked). The patients developed three Severe-IRIS-KS events and died.
(TIF)

S2 Fig Clinical evolution, CD4+ cell count, HIV, and HHV-8 viral load for each visit in a patient from the experimental group, with pulmonary KS diagnosed at baseline; he never developed Severe-IRIS-KS and had an uneventful evolution, with 70% remission at week 48. (TIF)

S3 Fig Patient in the EG that interrupted valganciclovir treatment on week 8, his HHV-8 VL rebounded, and on week 21 developed Severe-IRIS-KS. Valganciclovir was re-started on week 24; HHV-8 VL replication was finally suppressed. This subject was excluded from the per-protocol analysis.
(TIF)

S1 File (ZIP)

S2 File (PDF)

S3 File (PDF)

S4 File (DTA)

S5 File (PDF)
