# Supplementary figures and images for: Impact of valganciclovir therapy on severe IRIS-Kaposi Sarcoma mortality: An open-label, parallel, randomized controlled trial
Source: PLoS One. 2023 May 17;18(5):e0280209. doi: 10.1371/journal.pone.0280209 (PMC10191357; doi:10.1371/journal.pone.0280209)

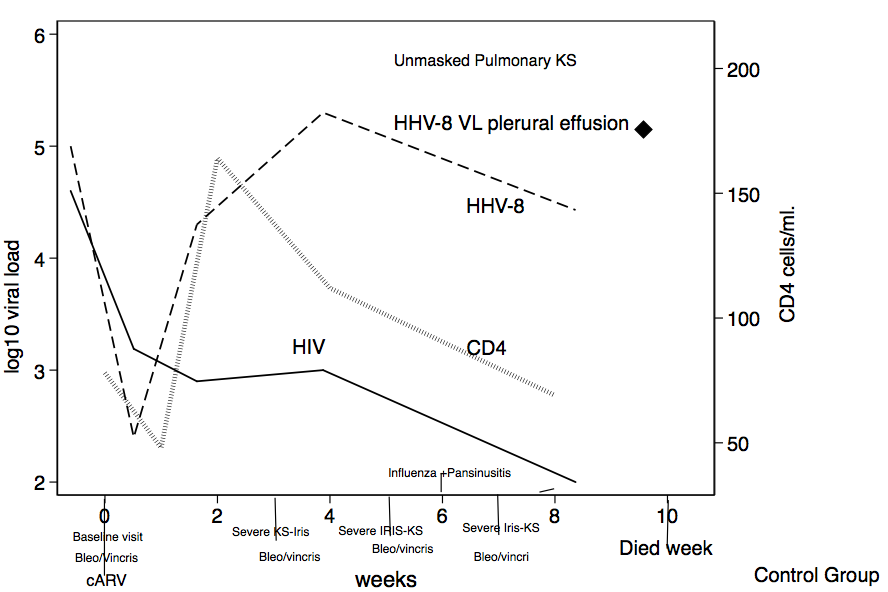

Supplement: S1 Fig — The patients developed three Severe-IRIS-KS events and died. (TIF) [file pone.0280209.s002.tif]

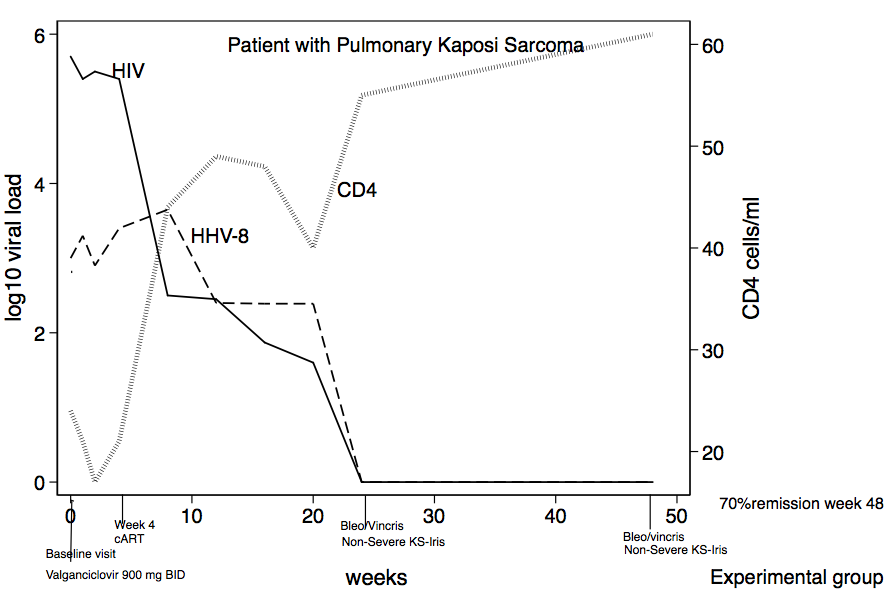

Supplement: S2 Fig — (TIF) [file pone.0280209.s003.tif]

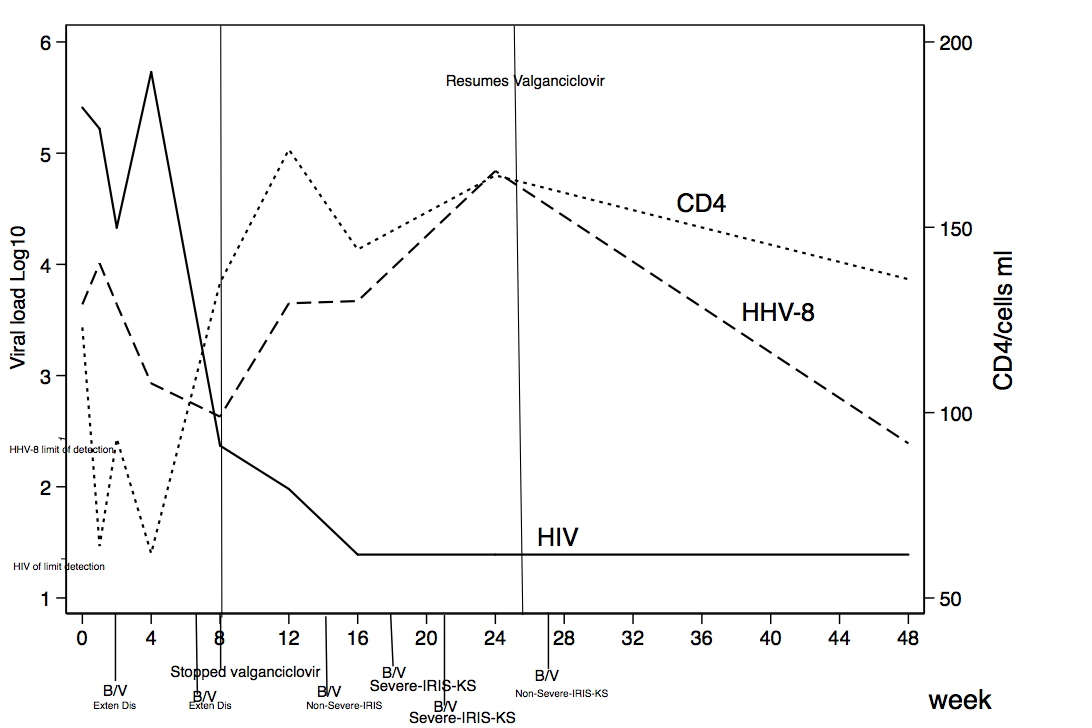

Supplement: S3 Fig — Valganciclovir was re-started on week 24; HHV-8 VL replication was finally suppressed. This subject was excluded from the per-protocol analysis. (TIF) [file pone.0280209.s004.tif]
