# Supplementary material for: Impact of valganciclovir therapy on severe IRIS-Kaposi Sarcoma mortality: An open-label, parallel, randomized controlled trial
Source: PLoS One. 2023 May 17;18(5):e0280209. doi: 10.1371/journal.pone.0280209 (PMC10191357; doi:10.1371/journal.pone.0280209)
Supplement: S1 File — (ZIP) [file pone.0280209.s005.zip › COI de autores/coi_disclosure ISLAS-MUN╠âOZ.pdf]

ICMJE DISCLOSURE FORM

Date:___June 20, 2021____
Your Name:__Beda Daniela Islas Muñoz_ ____
Manuscript Title:_ Impact of valganciclovir therapy on severe IRIS-Kaposi Sarcoma mortality: an open-label, parallel, randomized controlled-trial.
____
Manuscript number (if known):____

In the interest of transparency, we ask you to disclose all relationships/activities/interests listed below that are related to the content of your manuscript. "Related" means any relation with for-profit or not-for-profit third parties whose interests may be affected by the content of the manuscript. Disclosure represents a commitment to transparency and does not necessarily indicate a bias. If you are in doubt about whether to list a relationship/activity/interest, it is preferable that you do so.

The following questions apply to the author's relationships/activities/interests as they relate to the current manuscript only.

The author's relationships/activities/interests should be defined broadly. For example, if your manuscript pertains to the epidemiology of hypertension, you should declare all relationships with manufacturers of antihypertensive medication, even if that medication is not mentioned in the manuscript.

In item #1 below, report all support for the work reported in this manuscript without time limit. For all other items, the time frame for disclosure is the past 36 months.

		**Name all entities with whom you have this relationship or indicate none (add rows as needed)**	**Specifications/Comments (e.g., if payments were made to you or to your institution)**
**Time frame: Since the initial planning of the work**			
1	All support for the present manuscript (e.g., funding, provision of study materials, medical writing, article processing charges, etc.) **No time limit for this item.**	_X_ None	
**Time frame: past 36 months**			
2	Grants or contracts from any entity (if not indicated in item #1 above).	_X_ None	
3	Royalties or licenses	_X_ None	

4	Consulting fees	_X_ None	
5	Payment or honoraria for lectures, presentations, speakers bureaus, manuscript writing or educational events	_X_ None	
6	Payment for expert testimony	_X_ None	
7	Support for attending meetings and/or travel	_X_ None	
8	Patents planned, issued or pending	_X_ None	
9	Participation on a Data Safety Monitoring Board or Advisory Board	_X_ None	
10	Leadership or fiduciary role in other board, society, committee or advocacy group, paid or unpaid	_X_ None	
11	Stock or stock options	_X_ None	
12	Receipt of equipment, materials, drugs, medical writing, gifts or other services	_X_ None	
13	Other financial or non-financial interests	_X_ None	

Please place an "X" next to the following statement to indicate your agreement:

_X__ I certify that I have answered every question and have not altered the wording of any of the questions on this form.

Beda Daniela Islas Muñoz
