# Supplementary material for: Impact of valganciclovir therapy on severe IRIS-Kaposi Sarcoma mortality: An open-label, parallel, randomized controlled trial
Source: PLoS One. 2023 May 17;18(5):e0280209. doi: 10.1371/journal.pone.0280209 (PMC10191357; doi:10.1371/journal.pone.0280209)
Supplement: S1 File — (ZIP) [file pone.0280209.s005.zip › COI de autores/tlhiv-author-signatures.pdf]

THE LANCET HIV

Author statements

Please insert the relevant text under the subheadings below. A completed form must be signed by all authors. Please note that we will accept hand-signed and electronic (typewritten) signatures. Please complete multiple forms if necessary, and upload the signed copy with your submission, scan and email to: TheLancetHIV@lancet.com.

Manuscript title:

Corresponding author:

Article type:

I irrevocably authorise and grant my full consent to the corresponding author of the manuscript to: (1) enter into an exclusive publishing agreement with Elsevier on my behalf, in the relevant form set out at www.elsevier.com/copyright; and (2) unless I am a US government employee, to transfer my copyright or grant an exclusive license of rights to Elsevier as part of that publishing agreement, effective on acceptance of the article for publication. If the article is a work made for hire, I am authorized to confirm this on behalf of my employer. I agree that the copyright status selected by the corresponding author for the article shall apply and that this agreement is subject to the governing law of England and Wales.

Does your manuscript have a reference number? No ☐ Yes ☐ If yes, enter number here:

Does your manuscript have a handling editor? No ☐ Yes ☐ If yes, enter name here:

Authors' contributions

Please insert here the contribution each author made to the manuscript—eg, literature search, figures, study design, data collection, data analysis, data interpretation, writing etc. You may also find the CRediT taxonomy helpful. See http://credit.niso.org/ for more information on 14 roles that can be attributed to authors: conceptualisation, data curation, formal analysis, funding acquisition, investigation, methodology, project administration, resources, software, supervision, validation, visualisation, writing – original draft, and writing – review & editing. If all authors contributed equally, please state this. The information provided here must match the contributors' statement in the manuscript. We require that more than one author has verified the underlying data. Please state the named authors who have accessed verified the underlying data.

THE LANCET HIV

Role of the funding source

Please disclose any funding sources and their role, if any, in the writing of the manuscript or the decision to submit it for publication. Examples of involvement include: data collection, analysis, or interpretation; trial design; patient recruitment; or any aspect pertinent to the study. Please also comment whether you have been paid to write this article by a pharmaceutical company or other agency. The information provided here must match the role of the funding source statement in the manuscript. If you are the corresponding author please state that all authors had full access to the full data in the study and accept responsibility to submit for publication.

Conflicts of interest

Please complete the ICMJE conflict of interest form, which is available at http://download.thelancet.com/flatcontentassets/authors/icjme-coi-form.pdf. Please ensure that a conflict of interest statement is included at the end of the manuscript, which matches what is declared on the ICMJE conflict of interest form.

Patient consent (if applicable) - completion of this section is mandatory for Case Reports, Clinical Pictures, and Adverse Drug Reactions.
Please sign below to confirm that all necessary consents required by applicable law from any relevant patient, research participant, and/or other individual whose information is included in the article have been obtained in writing. **The signed consent form(s) should be retained by the corresponding author and NOT sent to *The Lancet HIV*.**

I agree with: the plan to submit to *The Lancet HIV;* the contents of the manuscript; to being listed as an author; and to the conflicts of interest statement as summarised. I have had access to all the data in the study (for original research articles) and accept responsibility for its validity.

Title and name:	Highest degree:	Signature:	Date:
Title and name:	Highest degree:	Signature:	Date:
Title and name:	Highest degree:	Signature:	Date:
Title and name:	Highest degree:	Signature:	Date:
Title and name:	Highest degree:	Signature:	Date:
Title and name:	Highest degree:	Signature:	Date:
Title and name:	Highest degree:	Signature:	Date:
Title and name:	Highest degree:	Signature:	Date:
Title and name:	Highest degree:	Signature:	Date:
Title and name:	Highest degree:	Signature:	Date:

Corresponding author declaration

I _______[Signature]_______ , the corresponding author of this manuscript, certify that the contributors' and conflicts of interest statements included in this paper are correct and have been approved by all co-authors.
