# Supplementary material for: Impact of valganciclovir therapy on severe IRIS-Kaposi Sarcoma mortality: An open-label, parallel, randomized controlled trial
Source: PLoS One. 2023 May 17;18(5):e0280209. doi: 10.1371/journal.pone.0280209 (PMC10191357; doi:10.1371/journal.pone.0280209)
Supplement: S2 File — (PDF) [file pone.0280209.s006.pdf]

Suppression of the HVH-8 viral load before the start of highly active antiretroviral therapy in patients with severe disseminated KS pulmonary and/ or extensive cutaneous and its impact on the development of Immune Recovery Syndrome (IRIS) and on the attributable mortality. Comparison with immediate start standard therapy (HAART). (Version 2- June 2, 2015)

Introduction: Kaposi's sarcoma (KS) is an angioproliferative disease associated with HIV infection, mediated by cytokines where the presence of human herpes virus 8 (HHV-8) and immunosuppression are essential for the process. It has an unpredictable course, it can be indolent or a progressive and fulminant course. Patients with disseminated KS have pulmonary disease and/or extensive skin disease and/or lymphadenopathic involvement, generally associated with low CD4 counts (<100 cells) and who may develop immune reconstitution syndrome (IRIS) after starting combined antiretroviral treatment (cART). Ganciclovir and foscarnet (antivirals) are active in vitro against HHV-8. Our group documented in a retrospective study with patients treated with ganciclovir for the management of cytomegalovirus (CMV) infection in pre-HAART that they developed complete remission. Patients with infections such as meningeal tuberculosis, cryptococcal meningitis or chorioretinitis due to CMV and low CD4, have a higher risk of developing Immune Recovery Syndrome (IRIS) and as a consequence of IRIS develop serious complications that can cause death or irreversible sequelae. In these cases, it has been shown that treating opportunistic infection and delaying the onset of cART, usually 4 weeks, avoids these complications.

Objective: To assess the decrease in mortality in patients with AIDS and severe IRIS-KS in patientes with (pulmonary and/or disseminated and/or lymphadenopathic and/or generalized lymphedema and /or involvement of the gasto-intestinal tract) with the use of ganciclovir or the prodrug valganciclovir before the initiation of HAART compared to HAART standard immediate start management.

Hypothesis: The administration of ganciclovir and the suppression of HHV-8 replication before the administration of HAART, with or without chemotherapy in patients with AIDS and disseminated KS, will decrease the frequency of IRIS and impact on reducing the mortality attributable to KS.

Methodology: Open randomized clinical trial. Patients with AIDS and severe KS who agree to participate and sign an informed consent letter will be included. A case of diseminated KS is considered when it presents one or more of the following: pulmonary involvement, and / or extensive cutaneous and/or lymphadenopathy and /or generalized lymphedema, involvement of the digestive tract documented in at least two segments oral cavity, esophagus , stomach or colon)
Patients with AIDS and synchronously presenting another neoplasm, receiving cortico-steroids, or replicative co-infection with hepatitis B or hepatitis C virus will be excluded. The study will consist of 2 study groups:
1. Group 1 (randomized): Patients with HIV and disseminated KS, who will receive ganciclovir treatment for four weeks prior to initiation of HAART and / or until

suppression of HHV-8 or twelve weeks (was extended to 48 weeks) 2. Group 2 (randomized): Patients with HIV and disseminated KS, who start standard treatment with HAART.

In all the groups, the need for QT will be managed at the discretion of the treating physician. The sample size was calculated for a power of 80% and an alpha of 0.05. Event rate in the control group 40%, and in the treated group 5%. The number of patients in each group will be 19 for a total sample of 38. Considering a perity of 20%, the sample size should be 46. An intermediate analysis will be carried out with in 20 patients with a follow-up of 48 weeks.

In all groups, the need for chemotherapy will be managed at the discretion of the treating physician.

The sample size was calculated for a power of 80% and an alpha of 0.05. Event rate in the control group 40%, and in the treated group 5%. The number of patients in each group will be 19 for a total sample of 38. Considering a perity of 20%, the sample size should be 46. An intermediate analysis will be carried out with in 20 patients with a follow-up of 48 weeks. . The antiretroviral regimen will be assigned according to the criteria of the current "Antiretroviral Management Guide for People living with HIV". A baseline clinical evaluation will be carried out to define the extension of KS (in the skin, at the gastrointestinal level through endoscopy and colonoscopy), at the lung and lymphatic level (chest CT in case of suspected lung involvement and PET in case of lymphadenopathy). Other active infections will be investigated and treated if necessary. IRIS criteria: patients with two weeks of HAART onset according to having one laboratory and two clinical criteria: Has a reduction of at least 1 log10 of HIV-1 viral load levels and ≥50 cells / mm3 increase or at least ≥2-fold increase in baseline CD4 + cell count KS exacerbation by at least two of the following clinical criteria: Increase in the size of KS lesions Increase in the number of KS lesions Onset or exacerbation of lymphedema The appearance or increase of otherwise unexplained pulmonary opacities on chest radiograph or gallium-negative chest CT.

The classification of the evolution of KS: Complete response (CR) when all KS lesions disappear Partial response (PR) when there was a> 50% decrease in the number and / or size of the original lesions without the appearance of new lesions, Stable disease (SD) was when lesions were reduced by <50% and no new lesions appeared. Disease progression (PD) when an increase in the number and size of KS lesions is documented in the follow-up periods. Relapse when new lesions appeared in a patient with CR or PR documented in previous evaluations. 10 mL of peripheral blood anticoagulated with EDTA will be obtained. This routine procedure will be performed by the patient's treating physician and a qualified phlebotomist. I
nflammation markers related to the response of known cytokines in KS will be measured at baseline and at weeks 1,2,4,8,12,16,24 and 48, such as: C-reactive protein, D-dimer, IL -6, Il-10, tumor necrosis factor (TNF). Serum and plasma will be obtained for HIV viral load, HVH-8 viral load, and complete blood count, CD4 lymphocyte count, liver function tests and blood chemistry will be performed. From the plasma sample, the absolute amount of each analyte will be quantified, we will use

the luminex methodology (Bio-Rad). The plasmas will be incubated with spheres coated with monoclonal antibodies directed against each analyte and on a specific fluorochrome, the fluorescence will be read on the Bio-Plex 2000 equipment (Bio-Rad). Absolute readings in μg / ml will be obtained for each analyte. The primary endpoint is the mortality attributable to KS at 24 weeks of the study,. Secondary outcomes: a) frequency and time of suppression of viral load of HHV-8 with ganciclovir, before and after initiation of HAART; b) frequency of IRIS at 48 weeks in patients treated with and without ganciclovir prior to starting HAART.

Introduction: Kaposi's sarcoma was described in the 19th century by Moritz Kaposi in older men from the Mediterranean area; [1] later it was recognized that this disease was endemic in Africa that could affect children, and in the seventies its frequency increased in patients with solid organ transplants and immunosuppression was recognized as a risk factor for the development of this [2]. In the early 1980s the occurrence of an unusually high number of this disease in young homosexuals was coupled with Pneumocystis jirovecci pneumonia, then called carinii, the marker for the onset of the AIDS epidemic [3-4]. Kaposi's Sarcoma was considered a low-grade neoplasm, however the epidemiology of the disease oriented towards an infectious agent, which was described in 1994 by the Dr. Chang group, since then the knowledge of this disease has increased and today it is recognized as an angioproliferative disease associated with HIV infection, mediated by cytosines where the presence of human herpes virus 8 (HHV-8) and immunosuppression is essential for the process [5,6,7].

The disease has an unpredictable course in both HIV-infected and uninfected people [8]. In the pre-HAART era (highly active antiretroviral therapy), patients could have an indolent course of the disease and die from another intercurrent problem or present with fulminant progressive disease and die from a cause attributable to Kaposi's Sarcoma (KS). Regarding treatment, patients who received the ABV chemotherapy regimens, which contain adriamycin, had a significantly lower survival than those who received (bleomycin-vincrsitin) or bleomycin alone, these are the two regimens that have been used in the last two decades at the National Cancer Institute [9].

The introduction in 1996, of the use of regimens combined with three antiretroviral drugs, made it possible for the first time to suppress the replication of the virus in the individual infected with HIV. These regimens are called Highly Active Antiretroviral Treatment (HAART and TARAA in Spanish). By suppressing the replication of HIV, the immune system recovers, and these schemes modified the course of AIDS, making it a chronic disease and dramatically decreasing the morbidity and mortality of HIV-infected patients who have access to these schemes. HAART schemes include two nucleoside analog reverse transcriptase inhibitors (ITRANs) with a non-nucleoside (NNRTI) or a protease inhibitor (PI) in which it is generally associated with an enhancer drug that is ritonavir. Although recently new groups of drugs have been included for the first line of treatment such as integrase inhibitors.

In the HAART era, between 9% and 50% of KS patients can achieve complete remission with HAART alone [10-18]. However, there is a subgroup of patients with a poor prognosis, mostly those with pulmonary involvement [19] or disseminated skin disease, mainly those who arrive with low CD4 and who develop immune reconstitution syndrome (IRIS). Patients with KS and low CD4 are those at higher risk of developing this complication, IRIS-KS, although some authors have found higher CD4 counts in patients with IRIS [20]. Patients with low CD4 are at greater risk of developing IRIS after the onset of HAART, with exacerbation of KS lesions, which was initially thought to be treatment failure and it was not until recently that it was recognized as a manifestation of IRIS in patients with KS. Mortality in this group of patients is 26%, the group with the highest mortality are patients with IRIS-KS and pulmonary involvement [17, 19,21]. The differentiation and proliferation of spindle cells is the result of multiple factors: host immunosuppression, cytokine production and probably the encoding of proteins that are homologous to human oncoproteins that regulate the different phases of cell growth; apoptosis and cytokine production [22-25]. Elevated levels of IL-6 and IL-10 have been described in this disease [26].

We have observed complete and sustained remission after ganciclovir therapy in AIDS patients with and without HAART, and with and without chemotherapy [29]. Ganciclovir is a drug with antiviral activity used to treat Cytomegalovirus infections. It is a 2 'deoxyguanosine nucleoside analog that acts by competitively inhibiting deoxyguanosine triphosphate (dGTP), used by the DNA polymerase of viruses for its replication, avoiding this process. Cytomegalovirus thymidine kinase metabolizes ganciclovir to the phosphorylated active substance ganciclovir and this is the substance that inhibits dGTP It has been widely used to treat Cytomegalovirus (CMV) chorioretinitis in AIDS patients, although this use has decreased since the advent of HAART [31]. It has also been used in other immunosuppressed patient populations such as bone marrow or stem cell transplantation and solid organs when patients suffer from CMV disease. Ganciclovir is administered exclusively intravenously as an infusion diluted in physiological solution to pass in 60 minutes every 12 hours. The pro-drug called valganciclovir was developed, it is absorbed in the digestive tract and is metabolized in the intestinal wall and at the liver level to ganciclovir [32]. The bioavailability of ganciclovir is 60% once it is administered orally. The dosage is 900 mg every 12 hours and the tablets are 450 mg each. Ganciclovir is eliminated by the kidneys through glomerular filtration and tubular secretion, requires dose adjustment when creatinine clearance falls below 60 at half the dose and a quarter when clearance is 10 to 24. In patients with less than 10 creatinine clearance should not be used. In patients with liver damage the need for dose adjustment is unknown. This is a drug with demonstrated teratogenic and carcinogenic potential in animals and produces inhibition of spermatogenesis in animals. The main adverse effects are anemia, neutropenia, granulocytopenia, and thrombocytopenia. Fever, nausea, vomiting, dyspepsia, diarrhea, anorexia, and increases in blood creatinine and urea levels have also been described less frequently. It interacts with some drugs increasing the risk of kidney damage such as aminoglycosides, carboplatin, probenecid, Acyclovir and bleomycin. With imipenem the risk of seizures produced by this second drug is increased. And associated with several antineoplastic drugs such

as cyclophosphamide, doxorubicin, bleomycin, dacarbazine, cytarabine and others since myelosuppression can be increased.

Additionally, a lower risk of developing KS has been described in AIDS patients receiving ganciclovir or foscarnet as CMV treatment or prophylaxis [26-28]. Furthermore, foscarnet and cidofovir have eradicated HHV-8 from patients with KS and HIV, and without HIV, suggesting that these antiviral agents have a therapeutic role in the management of KS [13,30,36]. The clinical benefit [37,38] of eradicating an infectious agent that induces cell proliferation is not exclusive to KS, it has also been described for Helicobacter pylori in a patient with MALT lymphoma (mucosa-associated lymphoid tissue lymphoma), where it has shown regression. This multi-modal approach can open new ways in the management of proliferative processes where an infectious agent plays a role in physiopathogenesis [39,40,41]. On the other hand, the benefit of delaying the initiation of HAART has been described in patients with serious opportunistic infections, even in patients with very low CD4 levels, in whom the risk of IRIS is high and the consequences of the immune response can produce irreversible sequelae. and even death, [42] where management against the opportunistic pathogen is first started and HAART is deferred at least 4 weeks [43-45]. The initial treatment for Kaposi's Sarcoma in patients with HIV is HAART. In a few cases some additional management is required, usually chemotherapy, at INCAN the Bleomcin + Vincristine scheme has been used for 20 years, since a decrease in survival was observed and an explosive evolution after the use of the adrimycin + bleomycin + vincritine regime. This is a disease where immunosuppression is part of the pathogenesis and the deleterious effect of certain agents such as liposomal doxorubicin on cellular immunity (low CD4 levels after use) has already been described, in addition to an increased risk of secondary neoplasms with the use of this drug. [46-47] This has generated controversy regarding the use or not of chemotherapy agents, particularly myelosupressives.

IV. REFERENCES

1. Barun M. Classics in oncology: Idiopathic multiple pigmented sarcoma of the skin by Kaposi. CA Cancer J Clin. 1982; 32:340-347.

2. Penn I. Kaposi's sarcoma in organ transplant recipients: report of 20 cases. Transplantation. 1979 Jan;27(1):8-11.

3. Friedman-Kien AE, Laubenstein LJ, Rubinstein P, Buimovici-Klein E, Marmor M, Stahl R, Spigland I, Kim KS, Zolla-Pazner S. Disseminated Kaposi's sarcoma in homosexual men. Ann Intern Med. 1982 Jun;96(6 Pt 1):693-700.

4. Gottlieb GJ, Ragaz A, Vogel JV, Friedman-Kien A, Rywlin AM, Weiner EA, Ackerman AB. A preliminary communication on extensively disseminated

Kaposi's sarcoma in young homosexual men. Am J Dermatopathol. 1981 Summer;3(2):111-4.

5. Chang Y, Cesarman E, Pessin MS, Lee F, Culpepper J, Knowles DM, Moore PS. Identification of herpesvirus-like DNA sequences in AIDS-associated Kaposi's sarcoma. Science 1994; 266 (5192):1865-9.

6. Chang Y. KSHV, Kaposi´s Sarcoma, and related lymphoproliferative disorders. In Parsonnet J. Microbes and Malignanciey. Infection as a cause of human cancers. Oxford University Press 1999. New York USA. pp 207-231.

7. Gantt S, Casper C. Human herpesvirus 8-associated neoplasms: the roles of viral replication and antiviral treatment. Curr Opin Infect Dis. 2011; 24: 295-301. doi: 10.1097/QCO.0b013e3283486d04.

8. Dezube BJ. Clinical presentation and natural history of AIDS-related Kaposi's sarcoma. Hematol Oncol Clin North Am 1996; 10:1023.

9. Volkow, P Jacquemin B, Zinser JW, Perez Padilla R. Remisión Completa de Sarcoma de Kaposi en la era pre-Terapia Antiretroviral Altamente Activa (TARAA) después del uso de ganciclovir para tratamiento de infección por Citomegalovirus. Sometido Febrero 2015 Trabajo de entrada a la Academia Nacional de Medicina.

10. Murphy M, Armstrong D, Sepkowitz KA, Ahkami RN, Myskowski PL. Regression of AIDS-related Kaposi's sarcoma following treatment with an HIV-1 protease inhibitor. AIDS 1997; 11: 261-2.

11. Aboulafia DM. Regression of acquired immunodeficiency syndrome-related pulmonary Kaposi's sarcoma after highly active antiretroviral therapy. Mayo Clin Proc 1998; 73: 439-43.

12. Bower M, Weir J, Francis N, Newsom-Davis T, Powles S, Crook T, Boffito M, Gazzard B, Nelson M. The effect of HAART in 254 consecutive patients with AIDS-related Kaposi's sarcoma. AIDS 2009; 23: 1701-6. doi: 10.1097/QAD.0b013e32832d080d.

13. Pellet C, Chevret S, Blum L, Gauvillé C, Hurault M, Blanchard G, Agbalika F, Lascoux C, Ponscarme D, Morel P, Calvo F, Lebbé C. Virologic and immunologic parameters that predict clinical response of AIDS-associated Kaposi's sarcoma to highly active antiretroviral therapy. J Invest Dermatol 2001; 117: 858-63. Erratum in: J Invest Dermatol 2002 Apr;118(4):741.

14. Dupont C, Vasseur E, Beauchet A, Aegerter P, Berthé H, de Truchis P, Zucman D, Rouveix E, Saiag P. Long-term efficacy on Kaposi's sarcoma of highly active

antiretroviral therapy in a cohort of HIV-positive patients. CISIH 92. Centre d'information et de soins de l'immunodéficience humaine. AIDS 2000; 14: 987-93.

15. Cattelan AM, Calabrò ML, De Rossi A, Aversa SM, Barbierato M, Trevenzoli M, Gasperini P, Zanchetta M, Cadrobbi P, Monfardini S, Chieco-Bianchi L. Long-term clinical outcome of AIDS-related Kaposi's sarcoma during highly active antiretroviral therapy. Int J Oncol 2005; 27: 779-85.

16. Asiimwe F, Moore D, Were W, Nakityo R, Campbell J, Barasa A, Mermin J, Kaharuza F. Clinical outcomes of HIV-infected patients with Kaposi's sarcoma receiving nonnucleoside reverse transcriptase inhibitor-based antiretroviral therapy in Uganda. HIV Med 2012; 13:166-71.

17. Volkow-Fernández P, Cornejo-Juarez P. Immune reconstitution inflammatory syndrome in patients with Kaposi´s sarcoma starting HAART in México. Mexico City - AIDS 2008: Abstract no. WEPE0205".

18. Borok M, et al. A pilot study of the effects of highly active antiretroviral therapy on Kaposi's sarcoma in Zimbabwe: Mexico City - AIDS 2008: Abstract no. THPE0216.

19. Palmieri C, Dhillon T, Thirlwell C, Newsom-Davis T, Young AM, Nelson M, Gazzard BG, Bower M. Pulmonary Kaposi sarcoma in the era of highly active antiretroviral therapy. HIV Med 2006; 7: 291-3.

20. Bower M, Nelson M, Young AM, Thirlwell C, Newsom-Davis T, Mandalia S, Dhillon T, Holmes, Gazzard BG, Stebbing J. Immune reconstitution inflammatory syndrome associated with Kaposi's sarcoma. J Clin Oncol 2005; 23: 5224-8.

21. Leidner RS, Aboulafia DM. Recrudescent Kaposi's sarcoma after initiation of HAART: a manifestation of immune reconstitution syndrome. AIDS Patient Care STDS 2005; 19: 635-44.

22. Nair BC, DeVico AL, Nakamura S, Copeland TD, Chen Y, Patel A, O'Neil T, Oroszlan S, Gallo RC, Sarngadharan MG. Identification of a major growth factor for AIDS-Kaposi's sarcoma cells as oncostatin M. Science. 1992 Mar 13;255(5050):1430-2.

23. Miles SA, Martínez-Maza O, Rezai A, Magpantay L, Kishimoto T, Nakamura S, Radka SF, Linsley PS. Oncostatin M as a potent mitogen for AIDS-Kaposi's sarcoma-derived cells. Science. 1992 Mar 13;255(5050):1432-4.

24. Cai J, Gill PS, Masood R, Chandrasoma P, Jung B, Law RE, Radka SF. Oncostatin-M is an autocrine growth factor in Kaposi's sarcoma. Am J Pathol 1994; 145: 74-9.

25. Chang Y. KSHV, Kaposi's sarcoma, and related lymphoproliferative disorders. In: Parsonnet J, Editor. Microbes and Malignancy. Infection As a Cause of Human Cancers. New York: Oxford University Press; 1999. pp. 207-231.

26. Uldrick TS, Wang V, O'Mahony D, Aleman K, Wyvill KM, Marshall V, Steinberg SM, Pittaluga S, Maric I, Whitby D, Tosato G, Little RF, Yarchoan R. An interleukin-6-related systemic inflammatory syndrome in patients co-infected with Kaposi sarcoma-associated herpesvirus and HIV but without Multicentric Castleman disease. Clin Infect Dis 2010; 51: 350-8.

27. Neyts J and Clerq E. Antiviral Drug Susceptibility of Human Herpesvirus. Antimicrob Agents Chemother 1997; 41: 2754-6.

28. Medveczky MM, Hovarth E, Lund T and Medveczky P. *In vitro* antiviral drug sensitivity of Kaposi´s sarcoma-associated herpes virus. AIDS 1997; 11: 1327-32.

29. Volkow P, Zinser J. Long-term remission of Kaposi´s sarcoma in AIDS patients after ganciclovir therapy. Proceedings of ASCO Vol 2000. No 2265 pag. 575.

30. Badiaga S, Parola Ph, Zandotti C and Brouqi Ph. Successful Treatment of Kaposi´s sarcoma with combination of antiviral drug therapy and chemotherapy: two case reports. Clin Infect Dis 1998; 27:1558-9.

31. Smee DF, Martin JC, Verheyden JP, Matthews TR. Anti-herpesvirus activity of the acyclic nucleoside 9-(1,3-dihydroxy-2-propoxymethyl)guanine. Antimicrob Agents Chemother. 1983 May;23(5):676-82.

32. Wiltshire J. Hirankarn S, Farrell C, Paya C, Pescovitz MD, Humar A, Dominguez E, Washburn K, Blumberg E, Alexander B, Freeman R, Heaton N; Valganciclovir Solid Organ Transplant Study Group. Pharmacokinetic profile of ganciclovir after its oral administration and from its prodrug, valganciclovir, in solid organ transplant recipients. Clin Pharmacokinet. 2005;44(5):495-507.

33. Glesby MJ, Hoover DR, Weng S et al. Use of antiherpes drugs and the risk of Kaposi´s sarcoma: data from Multicenter AIDS Cohort Study. J Infect Dis 1996; 173: 1477-80.

34. Mocroft A, Youle M, Gazzard B, Morcinek J, Halai R and Phillips AN. Anti-herpesvirus treatment and risk of Kaposi´s sarcoma in HIV infection. AIDS 1996; 10: 1101-5.

35. Robles R, Lugo D, Gee L, Jacobson MA. Effect of antiviral drugs used to treat cytomegalovirus end-organ disease on subsequent course of previously diagnosed Kaposi's sarcoma in patients with AIDS. J Acquir Immune Defic Syndr Hum Retrovirol 1999; 20: 34-8.

36. Gantt S, Casper C. Human herpesvirus 8-associated neoplasms: the roles of viral replication and antiviral treatment. Curr Opin Infect Dis 2011; 24: 295-301.

37. Verucchi G, Calza L, Trevisani F, Zambruni A, Tadolini M, Giuliani R, Manfredi R, Andreone P, Chiodo F, Bernardi M. Human herpesvirus-8-related Kaposi's sarcoma after liver transplantation successfully treated with cidofovir and liposomal daunorubicin. Transpl Infect Dis 2005; 7: 34-7.

38. Wotherspoon AC, Doglioni C, Diss TC et al. Regression of primary low-grade B-cell gastric lymphoma of mucosa-associated lymphoid tissue after eradication of *Helicobacter pylori.* Lancet 1993; 342: 575-77.

39. Bayerdörffer E, Neubauer A, Rudolph B, Thiede Ch, Lehn N, Eidt S, Stolte M. Regression of primary gastric lymphoma of mucosa-associated lymphoid tissue after cure of *Helicobacter pylori* infection. Lancet 1995; 345:1591-94.

40. Raderer M, Pfeffel F, Pohl G, Mannhalter C, Vlencak J, Chott A. Regression of colonic low grade B cell lymphoma of the mucosa associated lymphoid tissue type after eradication of *Helicobacter pylori*. Gut 2000; 46: 133-5.

41. Huang GC, Sheu BS, Tsao CJ, Lin XZ, Su IJ. Eradication of *Helicobacter pylori* results in regression of B-cell low grade gastric MALToma with evident B-symptoms. Hepatogastroenterology 1998; 45: 2464-7

42. Makadzange AT, Ndhlovu CE, Takarinda K, Reid M, Kurangwa M, Gona P, Hakim JG.
Early versus delayed initiation of antiretroviral therapy for concurrent HIV infection and cryptococcal meningitis in sub-saharan Africa. Clin Infect Dis 2010 Jun 1;50(11):153-8.

43. Ortega-Larrocea G, Espinosa E, Reyes-Terán G. Lower incidence and severity of cytomegalovirus-associated immune recovery uveitis in HIV-infected patients with delayed highly active antiretroviral therapy. AIDS. 2005 Apr 29;19(7):735-8.

44. Török ME, Yen NT, Chau TT, Mai NT, Phu NH, Mai PP, Dung NT, Chau NV, Bang ND, Tien NA, Minh NH, Hien NQ, Thai PV, Dong DT, Anh do TT, Thoa NT, Hai NN, Lan NN, Lan NT, Quy HT, Dung NH, Hien TT, Chinh NT, Simmons CP, de Jong M, Wolbers M. Farrar JJ. Timing of initiation of antiretroviral therapy in human
immunodeficiency virus (HIV)--associated tuberculous meningitis. Clin Infect Dis.

2011 Jun;52(11):1374-83.

45. Abdool Karim SS, Naidoo K, Grobler A, Padayatchi N, Baxter C, Gray AL, Gengiah T, Gengiah S, Naidoo A, Jithoo N, Nair G, El-Sadr WM, Friedland G, Abdool Karim Q. Integration of antiretroviral therapy with tuberculosis treatment. N Engl J Med. 2011 Oct 20;365(16):1492-501. doi: 10.1056/NEJMoa1014181.

46. Martín-Carbonero L, Palacios R, Valencia E, Saballs P, Sirera G, Santos I, Baldobí F, Alegre M, Goyenechea A, Pedreira J, González del Castillo J, Martínez-Lacasa J, Ocampo A, Alsina M, Santos J, Podzamczer D, González-Lahoz J; Caelyx/Kaposi's Sarcoma Spanish Group Long-term prognosis of HIV-infected patients with Kaposi sarcoma treated with pegylated liposomal doxorubicin. Clin Infect Dis. 2008 Aug 1;47(3):410-7.

47. Volkow P, Lizano M, Carrillo-García A, Pérez-Montiel D, Garciadiego P. Triple secondary neoplasms: penis, lip and oral cavity in an AIDS patient treated withpegylated liposomal doxorubicin for cutaneous Kaposi's sarcoma. AIDS. 2014 Sep 24;28(15):2327-9.
