# Supplementary material for: Impact of valganciclovir therapy on severe IRIS-Kaposi Sarcoma mortality: An open-label, parallel, randomized controlled trial
Source: PLoS One. 2023 May 17;18(5):e0280209. doi: 10.1371/journal.pone.0280209 (PMC10191357; doi:10.1371/journal.pone.0280209)
Supplement: S3 File — (PDF) [file pone.0280209.s007.pdf]

Valganciclovir four weeks prior to initiation of cART initiation compared to standard therap in patients with disseminated Kaposi.

Kaposi sarcoma (KS) is a Human Herpes-8 disease, it has been described as an angioproliferative disease mediated by cytokines in an environment of immunodeficiency and thus associated with HIV infection. Its course has been described as unpredictable; it can be indolent or can entertain a progressive and fulminant course. Patients with disseminated KS can have an extensive disseminated cutaneous disease with lymphedema and/or lymph node, gastrointestinal or pulmonary involvement, usually associated with low CD4 counts (<100 cells).
Higher early mortality in patient starting TARc has been described in different settings, and it has been attributed to the development of Immune Reconstitution Syndrome KS (IRIS-KS). In patients developing IRIS_KS what their life is not explained by the increase in KS lesion, lymphedema or pleural effusion that si associated with CD4 cells increase and the decrease of at least one log of HIV VL. The clinical picture of IRIS KS that threatens the patient's life is the abrupt development of fever with no other infection recognized, rapid clinical deterioration with thrombocytopenia, anemia, hyponatremia, and hypoalbuminemia together with an increase in KS lesion, lymphedema, or pleural effusion, this clinical picture we have named Severe-Iris-KS.
Patients with AIDS and disseminated KS are at risk of developing severe Immune Reconstitution Syndrome KS (S-IRIS-KS) after the initiation of cART and this has been associated with high mortality.
Ganciclovir and Foscarnet (antivirals) are active in vitro against HHV-8; valganciclovir is a prodrug of ganciclovir, it is an oral formulation. Our group documented, in a retrospective study that patients that were treated with ganciclovir for the management of Cytomegalovirus (CMV) end-organ disease in the pre-cART era achieve KS complete remission.

Our group documented, in a retrospective study that patients that were treated with ganciclovir for the management of Cytomegalovirus (CMV) end-organ disease in the pre-cART era achieve KS complete remission.

Patients with infections such as meningeal tuberculosis, cryptococcal meningitis, or CMV-related chorioretinitis, are also at a greater risk of developing IRIS, and as a consequence of it, deteriorate their clinical conditions that can even lead to death or irreversible sequel. In these cases, it has been demonstrated that starting treatment for the opportunistic infection and delays two or four weeks cART initiation; is possible to avoid or diminish these complications.

Objective:

To evaluate the occurrence of S-IRIS -KS and it attributable mortality in patients with HIV and disseminated KS (pulmonary and/or disseminated, and/or lymphadenopathic, and/or generalized lymphedema, and/or intestinal tract compromise) with the use of valganciclovir (ganciclovir prodrug, oral formulation) prior to the initiation of cART compared with the standard management of immediate cART initiation.

Hypothesis:

In patients with AIDS and disseminated KS administration of valganciclovir as an anti-HHV-8 agent can diminish viral replication prior to administration of cART, and thus decrease the frequency of S-IRIS-KS and will exert an impact on diminishing S-IRIS-KS attributable mortality.
Methodology:
Open randomized clinical assay. Will include patients with AIDS and disseminated KS who accept to participate and sign informed consent.

Inclusion criteria: Patients >18 years old, HIV+ naïve to cART with DKS, able and willing to provide written informed consent.
DKS was defined as the presence of KS pulmonary disease and/or ≥30 KS skin lesions, with or without lymphedema, and/or lymph node involvement, and/or GIT KS involvement (biopsy-proven at least in one site).
Exclusion criteria: Another concomitant malignancy, Multicentric Castleman Disease (MCD), steroid treatment two months prior to screening, active HBV or HCV or CMV end-organ disease, or severely ill patients with APACHE II score >15.
The study will comprise two study groups:

1. Group 1:Patients will receive treatment with valanciclovir during 4 weeks prior to initiation of cART and/or continue for 48 weeks
2. Group 2: Patients will initiate standard treatment with cART.

Both groups recieve bleomycin/vincristine; according to treating physician.
Patients will be randomized by blocks of ten; the assigned group written in closed envelopes: either to Experimental Group 1 (EG) to receive valganciclovir 900 mg twice daily for 48 weeks and initiate cART at week 4 after randomization.
Or to the Control Group 2(CG) to start cART immediately according to current Mexican Guidelines.

Sample size was calculated for a study power of 80% and an alpha of 0.05. Event rate in the control group was 40%, while that in the treated group was 5%. The number of patients in each group will be 19 for as total sample of 38 patients.
The antiretroviral scheme will be assigned according to the criteria of the "The Antiretroviral Management Guide of Persons with HIV" in effect in Mexico.

Candidates will have an initial thorough clinical evaluation including a work-up to diagnose coinfections and rule out other neoplasms that comprise: ophthalmologic evaluation, computed tomography (CT) scan (neck, thorax, and abdomen), bone marrow culture with bone biopsy, Hepatitis B Virus (HBV) and Hepatitis C Virus (HCV) serology, venereal disease research laboratory (VDRL) test and if indicated lumbar puncture to rule-out neurosyphilis, Histoplasma urinary antigen and serology, and GenXpert MTB/RIF test. Upper gastrointestinal tract (GIT) endoscopy with biopsy of lesions; colonoscopy will be performed only on patients with diarrhea or lower-GIT bleeding. Biopsies of enlarged lymph nodes will be obtained and process for histopathological analysis and culture. If indicated, bronchoscopy with bronchoalveolar lavage (BAL) will be performed as well as a thoracic Gallium 67 Spect-CT Scan or PET-FDG scans.
Criteria for Non-svere-IRIS KS and Severe-IRIS-KS

Non-severe-IRIS-KS: si defined as an increase in the number of KS lesions plus ≥one log10 of HIV-1 RNA VL decrease or ≥50 cells/mm3 or ≥2-fold from baseline CD4+ cells increase after cART initiation.

Severe -IRIS-KS is defined as an abrupt clinical deterioration after cART initiation alongside the presence of at least two clinical and at least three laboratory criteria.
Clinical criteria: 1) Fever (no identified concomitant infection), 2) increase in the size or number of KS lesions, 3) exacerbation of lymphedema, 4) appearance or increase of otherwise unexplained lung opacities on the chest images with a negative Gallium-Scan negative and 4) appearance or increase of pleural effusion.
Laboratory criteria: 1) Thrombocytopenia <100,000 platelets/ml; 2) Anemia (decrease of at least 1 g/dl from previous measure and no obvious bleeding), 3) Hyponatremia <135 mEq/L and 4) Hypoalbuminemia <3.5 g/dL. This is the clinical picture associated with death.
Classification of KS evolution:
Complete Response (CR) when all of the KS lesions disappear
Partial Response (PR) when there was a diminution of >50% in number and/or size of original lesions without the appearance of new lesions
Stable Disease (SD) was when there was a reduction of <50% of lesions and new lesions have not appeared
Disease Progression (DP) when an increase is documented of the number and size of the KS lesions during follow-up periods
Relapse when new lesions appeared in patients with CR or documented CR in previous evaluations.
Visits will be performed at baseline, week 1, 2, 4, 8, 12, 16, 24, and 48. At each visit, an Infectious Diseases specialist will perform a clinical evaluation, and pictures of skin lesions will photograph. Blood samples will be obtained for: WBC count, complete blood chemistry, urine analysis, CRP, D-Dimer, HIV VL with Abbott Real-time, HHV-8, CMV, and Epstein-Barr Virus (EBV) VL ELITe MGB KIT by ELITe InGenius Software, CD4+ and CD8+ cells count and percentage, (flow cytometry, Facs Canto II, Becton Dickinson) CD4/CD8 ratio and plasma levels of interleukin 6, 10 (IL-6, IL-10), tumor necrosis factor (TNF) and interferon-gamma (IFN-ɤ) were measured using a sandwich-type immunoassay, ELISA (Biolegend). Serology for syphilis, HBV, and HCV will be repeated at week 24 and 48. If patients had an exacerbation of KS outside the scheduled visit they will be reevaluated with laboratory tests and study images.
A qualified phlebotomist will carry out blood sampling.
