# Supplementary material for: Impact of valganciclovir therapy on severe IRIS-Kaposi Sarcoma mortality: An open-label, parallel, randomized controlled trial
Source: PLoS One. 2023 May 17;18(5):e0280209. doi: 10.1371/journal.pone.0280209 (PMC10191357; doi:10.1371/journal.pone.0280209)
Supplement: S5 File — (PDF) [file pone.0280209.s009.pdf]

FORMATO ÚNICO PARA EL REGISTRO DE PROTOCOLOS DE INVESTIGACIÓN

COMITÉ DE INVESTIGACIÓN Y ÉTICA INSTITUTO NACIONAL DE CANCEROLOGÍA

I. DATOS GENERALES

1. INVESTIGADOR PRINCIPAL

Nombre	Patricia Volkow Fernández
RFC:	VOFP571116/D0
Nacionalidad:	Mexicana
Cargo:	Médico especialista Dpto. de Infectología
Teléfono particular	
Teléfono de oficina	554747-1020 ext. 12110
Dirección de correo electrónico	pvolkowf@gmail.com
Nivel máximo de estudios:	Médico especialista
Disciplina	Infectología
Especialidad	Infectología
Categoría y nivel:	Médico especialista
Adscripción	Departamento de Infectología.

2. PROYECTO

Nombre del proyecto

Supresión de la carga viral del HVH-8 antes del inicio del *Tratamiento antirretroviral altamente activo (TARAA)* en pacientes con Sarcoma de Kaposi pulmonar y/o cutáneo diseminado grave y su impacto en el desarrollo de *Síndrome de recuperación inmune* (SIRI) y en la mortalidad atribuible. Comparación *con la terapia estándar (TARAA) de inicio inmediato.* (Versión 2- 2 de junio del 2015)

Tipo de investigación
(Marque con una X)

	Básica		Exploratoria
X	Clínica		Propositiva Experimental
	Mixta	X	Comparativa

Origen
(Marque con una X)

x	Interno
	Externo
	Tesis de Grado

Duración del proyecto

Inicio: __julio__/2015 Termino: _ene/2018
Mes Año Mes Año

Dr. José Luis Aguilar Ponce

Departamento: Infectología . Nombre del coordinado

3. COLABORADORES
(Se deberá anexar una carta de apoyo de cada colaborador en la que describa las actividades que realizará)

Colaborador I

Nombre:	Dra. Patricia Cornejo Juárez
RFC:	COJD700125Q12
Cargo:	Jefe Departamento de Infectología.
Nivel máximo de estudios:	Especialista en Infectología
Disciplina:	Infectología
Especialidad:	Infectología
Categoría y nivel:	Médico especialista
Adscripción:	Subdirección de Medicina Interna.

Colaborador II

Nombre:	Dra. Alexandra Martin-Onraët Arciniegas.
RFC:	MAAA811117N61
Cargo:	Médico especialista Dpto. Infectología
Nivel máximo de estudios:	Especialista en Infectología
Disciplina:	Infectología
Especialidad:	Infectología
Categoría y nivel:	Médico especialista
Adscripción:	Departamento de Infectología.

Colaborador III

Nombre:	María Isabel Sada Ovalle.
RFC:	SAOI720219
Cargo:	Jefe de Laboratorio de Inmunología Integrativa. (Determinación interleucinas)
Nivel máximo de estudios:	Post-doctorado.
Disciplina:	Inmunologia.
Especialidad:	Doctorado en Ciencias Biomédicas.
Categoría y nivel:	Investigador en Ciencias D.
Adscripción:	Instituto Nacional de Enfermedades Respiratorias.

Colaborador IV

Nombre:	Dra. Diana Vilar-Compte
RFC:	VICD661011MX9
Cargo:	Médico especialista
Nivel máximo de estudios:	Maestría
Disciplina:	Epidemiología
Especialidad:	Epidemiología hospitalaria
Categoría y nivel:	Médico Especialista.
Adscripción:	Departamento de Infectología

4. Colaborador V

Nombre:	Dra. Carolina Pérez Jiménez

RFC:	PEJC-780910-AG6
Cargo:	Médico especialista Dpto. Infectología
Nivel máximo de estudios:	Especialista en Infectología
Disciplina:	Infectología
Especialidad:	Infectología
Categoría y nivel:	Médico especialista
Adscripción:	Departamento de Infectología.

4. Colaborador VI

Nombre:	Dr. Juan Wolfang Zinser Sierra
RFC:	CURP: ZISJ501112HDFNRN03
Cargo:	Médico especialista Dpto. Oncología Médica
Nivel máximo de estudios:	Especialista en Oncología Médica
Disciplina:	Oncología Médica
Especialidad:	Oncología Médica
Categoría y nivel:	Médico especialista
Adscripción:	Departamento de Oncología Médica

OTRAS INSTITUCIONES PARTICIPANTES

(Se deberá anexar una carta de apoyo firmada por el director de cada instituto participante)

Nombre Institución I	Instituto Nacional de Enfermedades Respiratorias.
Nombre Institución II	
Nombre Institución III	
Nombre Institución IV	
Nombre Institución V	

Tipo de apoyo
(Marque con una X el tipo de ayuda proporcionado)

	Tipo de ayuda			
	Infraestructura	Personal	Material	Equipo
Nombre Institución I	X			
Nombre Institución II				
Nombre Institución III				
Nombre Institución IV				
Nombre Institución V				

II. SINTESIS DEL PROYECTO

Introducción:

El sarcoma de Kaposi (SK) es una enfermedad angioproliferativa asociada a infección por VIH, mediada por citosinas donde la presencia del herpes virus humano 8 (HVH-8) y la inmunosupresión son esenciales para el proceso. Tiene un curso impredecible, puede ser indolente o un curso progresiva y fulminante. Los pacientes con SK grave tiene afección pulmonar y/o,

enfermedad cutánea diseminada y/o involucro linfadenopático, generalmente asociado con cuentas bajas de CD4 (<100 células) y que puede desarrollar síndrome de reconstitución inmune (SIRI) posterior al inicio del tratamiento antirretroviral altamente activo (TARAA).
El ganciclovir y foscarnet (antivirales) son activos *in vitro* contra HVH-8. Nuestro grupo documentó en un estudio retrospectivo con pacientes tratados con ganciclovir para manejo de infección por citomegalovirus (CMV) en la preTARAA que desarrollaban remisión completa
Los pacientes con infecciones como tuberculosis meníngea, meningitis por criptococo o corioretinitis por CMVy CD4 bajos, tienen mayor riesgo de desarrollar *Síndrome de recuperación inmune* (SIRI) y como consecuencia del SIRI desarrollar complicaciones graves que causen la muerte a o secuelas irreversibles. En estos casos se ha demostrado que tratar la infección oportunista y diferir el inicio del TARAA, usualmente 4 semanas evita estas complicaciones.

Objetivo:
Evaluar la disminución en la mortalidad en pacientes con SIDA y SK grave (pulmonar y/o diseminado y/o linfadenopático y/o linfedema generalizado y/o compromiso del tracto intestinal) con el uso de ganciclovir o la prodroga valganciclovir antes del inicio de TARAA comparados con el manejo estándar de inicio inmediato de TARAA.

Hipótesis:
La administración de ganciclovir y la supresión de la replicación de HVH-8 antes de la administración de TARAA, con o sin quimioterapia en pacientes con SIDA y SK diseminado, disminuirá la frecuencia de SIRI e impactará en disminuir la mortalidad atribuible a SK.

Metodología:

Ensayo clínico aleatorizado abierto. Se incluirán pacientes con SIDA y SK grave que acepten participar y firmen carta de consentimiento informado.

Se considerar un caso de SK de grave cuando presente uno a o mas de los siguientes involucros: compromiso pulmonar, y/o cutáneo diseminado y/o linfadenopático y/o linfedema generalizado, compromiso de tracto digestivo documentado en al menos dos segmentos cavidad oral, esófago, estómago, intestino grueso)
Se excluirán pacientes con SIDA y que presenten en forma sincrónica otra neoplasia, que reciban cortico-esteroides, o co-infección replicativa con virus de hepatitis B o hepatitis C.

El estudio constará de 2 grupos de estudio y un tercer observacional:

1. Grupo 1 (aleatorizado): Pacientes con VIH y SK diseminado, que recibirán tratamiento con ganciclovir durante cuatro semanas previo al inicio de TARAA y/o hasta supresión de HVH-8 o doce semanas (lo que suceda primero).
2. Grupo 2 (aleatorizado): Pacientes con VIH y SK diseminado, que inician tratamiento estándar con TARAA.
3. Grupo Observacional : Pacientes con VIH y SK en quienes se documenta infección por CMV, y que requieren de inicio de ganciclovir al menos dos semanas previo al inicio de TARAA.

En los 3 grupos, la necesidad de QT se administrará a discreción del médico tratante.

El tamaño de la muestra se calculó para un poder del 80% y un alfa de 0.05. Tasa de eventos en el grupo control 40%, y en el grupo tratado de 5%. El número de pacientes en cada grupo será de 19 para una muestra total de 38. Considerando un peridad de 20%, el tamaño de la muestra deberá ser de 46. Se hará un anlisis intermedio al tener i 20 pacientes con un seguimiento de 24 semanas.

El esquema antiretroviral se asignará de acuerdo a los criterios de la *"Guía de Manejo Antirretroviral de las Personas con VIH" vigente.*

Se realizará una evaluación clínica basal para definir la extensión del SK (en piel, a nivel gastrointestinal por medio de endoscopia y colonoscopia), a nivel pulmonar y linfático (TAC tórax en caso de sospecha de compromiso pulmonar y PET en caso de linfadenopático). Se investigará otras infecciones activas y se dará tratamiento en caso necesario..

Criterios de SIRI: pacientes con dos semanas de inicio de TARAA de acuerdo que presenten un criterio de laboratorio y dos clínicos:

Tiene una reducción de al menos 1 log10 del VIH-1 los niveles de carga viral y

Aumento de ≥50 células / mm3 o un incremento de al menos ≥2 veces en los niveles basales de la cuenta de células CD4 +

Exacerbación del SK por al menos dos de los siguientes criterios clínicos:

Aumento en el tamaño de las lesiones de SK

Aumento el número de lesiones de SK

Aparición o exacerbación del linfedema

La aparición o aumento de las opacidades pulmonares de otro modo inexplicables en la radiografía de tórax o TC de tórax con estudio de galio negativo.

La clasificación de la evolución de KS :

Respuesta completa (RC) cuando desaparezcan todas las lesiones de SK

Respuesta parcial (RP) cuando hubo exista una disminución> 50% en el número y / o tamaño de las lesiones originales sin aparición de nuevas lesiones,

Enfermedad estable (EE) fue cuando se reduzcan en un <50% en las lesiones y no hayan aparecido nuevas lesiones.

Progresión de la enfermedad (PE) cuando se documente un aumento el número y tamaño de lesiones del SK en los períodos de seguimiento

Recaída cuando aparecieron nuevas lesiones en paciente con RC o RC documentado en la evaluaciones anteriores.

Se obtendrán 10 mL de sangre periférica anticoagulada con EDTA. Este procedimiento rutinario se realizará por el médico tratante del paciente y un flebotomista calificado.

Se medirán en forma basal y en las semanas 1,2,4,8,12,16,24 y 48, marcadores de inflamación relacionados con la respuesta de citosinas conocidas en SK, como son: proteína C reactiva, Dímero-D, IL-6, Il-10, factor de necrosis tumoral (FNT). Se obtendrá suero y plasma para carga viral de VIH, carga viral de HVH-8, y se realizará biometría hemática completa, cuenta de linfocitos CD4, pruebas de función hepática y química sanguínea. A partir de la muestra de plasmas se cuantificará la cantidad absoluta de cada analito, utilizaremos la metodología luminex (Bio-Rad). Los plasmas se incubaran con esferas recubiertas con anticuerpos monoclonales dirigidos contra cada analito y el un fluorocromo específico, la fluorescencia se leerá en el equipo Bio-Plex 2000 (Bio-Rad). Se obtendrán lecturas absolutas en µg/ml para cada análito.

El desenlace primario es la mortalidad atribuible a SK a las 24 semanas del estudio, en los tres grupos. Los desenlaces secundarios:

a) frecuencia y tiempo de supresión de carga viral de HVH-8 con ganciclovir, pre y post inicio de TARAA;

b) frecuencia de SIRI a las 24 semanas en pacientes tratados con y sin ganciclovir previo al inicio de HAART.

III. ANTECEDENTES DEL PROYECTO

Introducción:

El sarcoma de Kaposi fue descrita en el siglo XIX por Moritz Kaposi en hombres mayores del zona del mediterránea; [1] mas tarde se reconoció que esta enfermedad era endémica en África que podía afectar niño, y en los setentas su frecuencia se incremento en pacientes con trasplante de órganos sólidos y se reconoció a la inmunosupresión como un factor de riesgo para el desarrollo de esta [2]. A principios de la década de los ochenta la ocurrencia de un número inusualmente alto de esta enfermedad en jóvenes homosexuales fue junto con la neumonía por *Pneumocistis jirovecci*, entonces llamado *carinii*, el marcador del inicio de la epidemia de Sida [3-4]. El Sarcoma de Kaposi fue considero un neoplasia de bajo grado, sin embargo la epidemiología de la enfermedad orientaba hacia un agente infeccioso, el cual fue descrito en 1994 por el grupo Dr. Chang, a partir de entonces el conocimiento de esta enfermedad e ha incrementado y hoy se reconoce como una enfermedad angioproliferativa asociada a infección por VIH, mediada por citosinas donde la presencia del herpes virus humano 8 (HVH-8) y la inmunosupresión es esencial para el proceso [5,6,7].

La enfermedad tiene un curso impredecible tanto en pacientes infectados con VIH como en personas no infectadas [8]. En la era pre-TARAA (tratamiento antirretroviral altamente activo), los pacientes podían tener un curso indolente de la enfermedad y morir por otro problema intercurrente o presentarse con una enfermedad progresiva fulminante y morir por causa atribuible al Sarcoma de Kaposi (SK). En relación al tratamiento, los pacientes que recibían el esquemas de quimioterapia ABV, que contiene adriamicina, tenían una sobrevida significativamente menor que aquellos que recibían (bleomicina-vincrsitina) o bleomicina sola, estos son los dos esquemas que se han utilizada en las últimas dos décadas en el Instituto Nacional de Cancerología [9].

La introducción en 1996, del uso de esquemas combinados con tres fármacos antiretrovirales, permitió por primera vez suprimir la replicación del virus en el individuo infectado con VIH. A estos esquemas se les denomina Tratamiento Antiretroviral Altamente Activo (siglas en inglés HAART y en español TARAA). AL suprimir la replicación del VIH, el sistema inmune se recupera, y estos esquemas modificaron el curso del SIDA, volviendo esta una enfermedad crónica y disminuyendo dramáticamente la morbilidad y la mortalidad de los pacientes infectados con VIH que tienen accesos a estos esquemas. Los esquemas TARAA incluyen dos inhibidores de la transcriptasa reversa análogos nucleósidos (ITRANs) con un no nucleósido (ITRNN) o un inhibidor de proteasa (IP) en cual generalmente esta asociado a un medicamento potenciador que es el ritonavir. Aunque recientemente se han incluido grupo s nuevos de medicamentos para primera línea del tratamiento como lo son los inhibidores de integrasa.

En la era TARAA, entre 9 y 50% de los pacientes con SK pueden alcanzar remisión completa solo con TARAA [10-18]. Sin embargo existe un subgrupo de pacientes con mal pronóstico, en su mayoría aquellos con compromiso pulmonar [19] o enfermedad cutánea diseminada, principalmente aquellos que llegan con CD4 bajos y que desarrollan síndrome de reconstitución inmune (SIRI). Los pacientes con SK y CD4 bajos son los que tiene mayor riesgo de desarrollar esta complicación con SIRI-SK, aunque algunos autores han encontrado cuentas altas de CD4 en pacientes que SIRI en pacientes con SK [20]. Los pacientes con CD4 bajos tienen mayor riesgo de desarrollar SIRI después del inicio de TARAA, con exacerbación de las lesiones de SK, que en un inicio se pensó se

trataba de falla al tratamiento y no es hasta fecha reciente que se reconoció como una manifestación de SIRI en pacientes con SK. En este grupo de pacientes la mortalidad es de 26%, el grupo con mayor mortalidad son los pacientes con SIRI-SK y compromiso pulmonar [17, 19,21].

La diferenciación y proliferación de la células fusiformes ("*spindle cells"*) es el resultado de múltiples factores: inmunosupresión del hospedero, producción de citosinas y probablemente la codificación de proteínas que son homólogas a oncoproteínas humanas que regulan las diferentes fases del crecimiento de las células, la apoptosis y la producción de citosinas [22-25]. Niveles elevados de IL-6 y de IL-10 han sido descritos en esta enfermedad [26].

Como en todas las enfermedades de etiología infecciosa, la quimioterapia contra el agente patógeno parecerá adecuado para el tratamiento; el ganciclovir y el foscarnet han demostrado actividad in vitro contra HHV-8, e *in vivo* han demostrado erradicar el virus [27-28].

La observación retrospectiva de remisión prolongada y sostenida del SK en pacientes con SIDA después de la administración de ganciclovir para tratamiento de enfermedad por Citomegalovirus (CMV), es un indicador del posible papel terapéutico de este antiviral en el manejo del SK [29-30], particularmente para pacientes con enfermedad diseminada donde se han descrito carga virales altas de HVH-8 asociados con la gravedad de la enfermedad [26].

Nosotros hemos observado remisión completa y sostenida después de la terapia con ganciclovir en pacientes con SIDA con y sin TARAA, y con y sin quimioterapia [29].

El **ganciclovir** es medicamento con actividad antiviral utilizado para tratar infecciones por Citomegalovirus. Es un análogo nucleósido 2' desoxiguanosina que actúa inhibiendo de forma competitiva desoxiguanosina trifosfato (dGTP), utilizada por la DNA polimerasa de los virus para su replicación evitando se de este proceso. La timidin-quinasa del citomegalovirus metaboliza el ganciclovir a la sustancia activa ganciclovir fosforilado y este es la sustancia que inhibe la dGTP
Se ha utilizado ampliamente para tratar corioretinitis por Citomegalovirus (CMV) en pacientes con SIDA, si bien desde el advenimiento de la TARAA su uso se ha disminuido [31]. Se ha utilizado también en otras poblaciones de pacientes inmuno-suprimidos como son los de trasplante de médula ósea o células tronco y órganos sólidos cuando los pacientes sufren enfermedad por CMV. El ganciclovir se administra exclusivamente por vía intravenosa en infusión diluida en solución fisiológica a pasar en 60 minutos cada 12 horas. Fue desarrollado la pro-droga llamada valganciclovir, la se absorbe en el tubo digestivo y es metabolizada en la pared del intestino y a nivel del hígado a ganciclovir [32]. La biodisponibilidad del ganciclovir es del 60% una vez que se administra pro vía oral. La posología es de 900 mg cada 12 horas y los comprimidos son de 450 mg cada uno.
El ganciclovir se elimina pro vía renal a través de filtración glomerular y secreción tubular, requiere ajuste de la dosis cuando la depuración de creatinina cae por debajo de 60 a la mitad de la dosis y a una cuarta parte cuando la depuración es 10 a 24. En pacientes con menos de 10 de depuración de creatinina no debe ser utilizado. En pacientes con daño hepático no se conoce la necesidad de ajustar la dosis.
Este es un fármaco con potencial teratogénico y carcinogénico demostrado en animales y produce inhibición de la espermatogénesis en animales. Los principales efectos adversos son anemia, neutropenia, granulocitopenia y trombocitopenia. También se han descrito en menor frecuencia fiebre, náuseas, vómito, dispepsia, diarrea, anorexia e incrementos de los niveles de creatinina y urea en sangre.
Interactúa con algunos fármacos potenciando el riesgo de daño renal como son aminoglucósidos, carboplatino, probenecid, Aciclovir y bleomicina. Con el imipenem se incrementa el riesgo de convulsiones producidas por este segundo fármaco. Y asociado a varios fármacos antineoplásicos

como ciclofosfamida, doxorrubicina, bleomicina, dacarbacina, citarabina y otros ya que se puede incrementar la mielosupresión.

Adicionalmente se ha descrito un menor riesgo de desarrollar SK en pacientes con SIDA que reciben ganciclovir o foscarnet como tratamiento o profilaxis de CMV [26-28]. Aun más, el foscarnet y el cidofovir han erradicado el HVH-8 de pacientes con SK y VIH, y sin VIH, lo que sugiere que estos agentes antivirales tienen un papel terapéutico en el manejo del SK [13,30,36].

El beneficio clínico [37,38] de erradicar un agente infeccioso que induce la proliferación celular no es exclusive del SK, se ha descrito también para el *Helicobacter pylori* en paciente con linfoma de MALT (linfoma de tejido linfoide asociado a mucosa), donde se ha observado regresión. Este abordaje multi-modal puede abrir nuevas vías en el manejo de procesos proliferativos donde un agente infeccioso juego un papel en la fisiopatogénesis [39,40,41].

Por otra parte se ha descrito el beneficio de diferir el inicio de TARAA en pacientes con infecciones oportunistas graves, aun en pacientes con niveles muy bajos de CD4, en quienes el riesgo de SIRI es elevado y las consecuencias de la respuesta inmune puede producir secuelas irreversibles e inclusive a muerte, [42] donde primero se inicia el manejo contra el patógeno oportunista y TARAA se difiere al menos 4 semanas [43-45].

El tratamiento inicial del Sarcoma de Kaposi en pacientes con VIH es TARAA. En pocos casos se requiere de algún manejo adicional usualmente quimioterapia, en el INCAN se ha utilizado desde hace 20 años el esquema Bleomcina+Vincristina, ya que se observo un decremento en la sobrevida y un evolución explosiva posterior al uso del esquema adrimicina+bleomicjna+vincritina. Esta es una enfermedad donde la inmunosupresión es parte de la patogénesis y ya se ha descrito el efecto deletéreo de ciertos agentes como la doxorrubicina liposomal en la inmunidad celular (niveles bajos de CD4 después de su uso) además de un riesgo incrementado de ocurrencia de neoplasias secundarias con el uso de este fármaco. [46-47] Esto ha generado controversia en relación al el uso o no de agentes de quimioterapia en particular los mielosupressivos.

IV. REFERENCIAS

1. Barun M. Classics in oncology: Idiopathic multiple pigmented sarcoma of the skin by Kaposi. CA Cancer J Clin. 1982; 32:340-347.

2. Penn I. Kaposi's sarcoma in organ transplant recipients: report of 20 cases. Transplantation. 1979 Jan;27(1):8-11.

3. Friedman-Kien AE, Laubenstein LJ, Rubinstein P, Buimovici-Klein E, Marmor M, Stahl R, Spigland I, Kim KS, Zolla-Pazner S. Disseminated Kaposi's sarcoma in homosexual men. Ann Intern Med. 1982 Jun;96(6 Pt 1):693-700.

4. Gottlieb GJ, Ragaz A, Vogel JV, Friedman-Kien A, Rywlin AM, Weiner EA, Ackerman AB. A preliminary communication on extensively disseminated Kaposi's sarcoma in young homosexual men. Am J Dermatopathol. 1981 Summer;3(2):111-4.

5. Chang Y, Cesarman E, Pessin MS, Lee F, Culpepper J, Knowles DM, Moore PS. Identification of herpesvirus-like DNA sequences in AIDS-associated Kaposi's sarcoma. Science 1994; 266 (5192):1865-9.

6. Chang Y. KSHV, Kaposi´s Sarcoma, and related lymphoproliferative disorders. In Parsonnet J. Microbes and Malignanciey. Infection as a cause of human cancers. Oxford University Press 1999. New York USA. pp 207-231.

7. Gantt S, Casper C. Human herpesvirus 8-associated neoplasms: the roles of viral replication and antiviral treatment. Curr Opin Infect Dis. 2011; 24: 295-301. doi: 10.1097/QCO.0b013e3283486d04.

8. Dezube BJ. Clinical presentation and natural history of AIDS-related Kaposi's sarcoma. Hematol Oncol Clin North Am 1996; 10:1023.

9. Volkow, P Jacquemin B, Zinser JW, Perez Padilla R. Remisión Completa de Sarcoma de Kaposi en la era pre-Terapia Antiretroviral Altamente Activa (TARAA) después del uso de ganciclovir para tratamiento de infección por Citomegalovirus. Sometido Febrero 2015 Trabajo de entrada a la Academia Nacional de Medicina.

10. Murphy M, Armstrong D, Sepkowitz KA, Ahkami RN, Myskowski PL. Regression of AIDS-related Kaposi's sarcoma following treatment with an HIV-1 protease inhibitor. AIDS 1997; 11: 261-2.

11. Aboulafia DM. Regression of acquired immunodeficiency syndrome-related pulmonary Kaposi's sarcoma after highly active antiretroviral therapy. Mayo Clin Proc 1998; 73: 439-43.

12. Bower M, Weir J, Francis N, Newsom-Davis T, Powles S, Crook T, Boffito M, Gazzard B, Nelson M. The effect of HAART in 254 consecutive patients with AIDS-related Kaposi's sarcoma. AIDS 2009; 23: 1701-6. doi: 10.1097/QAD.0b013e32832d080d.

13. Pellet C, Chevret S, Blum L, Gauvillé C, Hurault M, Blanchard G, Agbalika F, Lascoux C, Ponscarme D, Morel P, Calvo F, Lebbé C. Virologic and immunologic parameters that predict clinical response of AIDS-associated Kaposi's sarcoma to highly active antiretroviral therapy. J Invest Dermatol 2001; 117: 858-63. Erratum in: J Invest Dermatol 2002 Apr;118(4):741.

14. Dupont C, Vasseur E, Beauchet A, Aegerter P, Berthé H, de Truchis P, Zucman D, Rouveix E, Saiag P. Long-term efficacy on Kaposi's sarcoma of highly active antiretroviral therapy in a cohort of HIV-positive patients. CISIH 92. Centre d'information et de soins de l'immunodéficience humaine. AIDS 2000; 14: 987-93.

15. Cattelan AM, Calabrò ML, De Rossi A, Aversa SM, Barbierato M, Trevenzoli M, Gasperini P, Zanchetta M, Cadrobbi P, Monfardini S, Chieco-Bianchi L. Long-term clinical outcome of AIDS-related Kaposi's sarcoma during highly active antiretroviral therapy. Int J Oncol 2005; 27: 779-85.

16. Asiimwe F, Moore D, Were W, Nakityo R, Campbell J, Barasa A, Mermin J, Kaharuza F. Clinical outcomes of HIV-infected patients with Kaposi's sarcoma receiving nonnucleoside

reverse transcriptase inhibitor-based antiretroviral therapy in Uganda. HIV Med 2012; 13:166-71.

17. Volkow-Fernández P, Cornejo-Juarez P. Immune reconstitution inflammatory syndrome in patients with Kaposi´s sarcoma starting HAART in México. Mexico City - AIDS 2008: Abstract no. WEPE0205".

18. Borok M, et al. A pilot study of the effects of highly active antiretroviral therapy on Kaposi's sarcoma in Zimbabwe: Mexico City - AIDS 2008: Abstract no. THPE0216.

19. Palmieri C, Dhillon T, Thirlwell C, Newsom-Davis T, Young AM, Nelson M, Gazzard BG, Bower M. Pulmonary Kaposi sarcoma in the era of highly active antiretroviral therapy. HIV Med 2006; 7: 291-3.

20. Bower M, Nelson M, Young AM, Thirlwell C, Newsom-Davis T, Mandalia S, Dhillon T, Holmes, Gazzard BG, Stebbing J. Immune reconstitution inflammatory syndrome associated with Kaposi's sarcoma. J Clin Oncol 2005; 23: 5224-8.

21. Leidner RS, Aboulafia DM. Recrudescent Kaposi's sarcoma after initiation of HAART: a manifestation of immune reconstitution syndrome. AIDS Patient Care STDS 2005; 19: 635-44.

22. Nair BC, DeVico AL, Nakamura S, Copeland TD, Chen Y, Patel A, O'Neil T, Oroszlan S, Gallo RC, Sarngadharan MG. Identification of a major growth factor for AIDS-Kaposi's sarcoma cells as oncostatin M. Science. 1992 Mar 13;255(5050):1430-2.

23. Miles SA, Martínez-Maza O, Rezai A, Magpantay L, Kishimoto T, Nakamura S, Radka SF, Linsley PS. Oncostatin M as a potent mitogen for AIDS-Kaposi's sarcoma-derived cells. Science. 1992 Mar 13;255(5050):1432-4.

24. Cai J, Gill PS, Masood R, Chandrasoma P, Jung B, Law RE, Radka SF. Oncostatin-M is an autocrine growth factor in Kaposi's sarcoma. Am J Pathol 1994; 145: 74-9.

25. Chang Y. KSHV, Kaposi's sarcoma, and related lymphoproliferative disorders. In: Parsonnet J, Editor. Microbes and Malignancy. Infection As a Cause of Human Cancers. New York: Oxford University Press; 1999. pp. 207-231.

26. Uldrick TS, Wang V, O'Mahony D, Aleman K, Wyvill KM, Marshall V, Steinberg SM, Pittaluga S, Maric I, Whitby D, Tosato G, Little RF, Yarchoan R. An interleukin-6-related systemic inflammatory syndrome in patients co-infected with Kaposi sarcoma-associated herpesvirus and HIV but without Multicentric Castleman disease. Clin Infect Dis 2010; 51: 350-8.

27. Neyts J and Clerq E. Antiviral Drug Susceptibility of Human Herpesvirus. Antimicrob Agents Chemother 1997; 41: 2754-6.

28. Medveczky MM, Hovarth E, Lund T and Medveczky P. *In vitro* antiviral drug sensitivity of Kaposi´s sarcoma-associated herpes virus. AIDS 1997; 11: 1327-32.

29. Volkow P, Zinser J. Long-term remission of Kaposi´s sarcoma in AIDS patients after ganciclovir therapy. Proceedings of ASCO Vol 2000. No 2265 pag. 575.

30. Badiaga S, Parola Ph, Zandotti C and Brouqi Ph. Successful Treatment of Kaposi´s sarcoma with combination of antiviral drug therapy and chemotherapy: two case reports. Clin Infect Dis 1998; 27:1558-9.

31. Smee DF, Martin JC, Verheyden JP, Matthews TR. Anti-herpesvirus activity of the acyclic nucleoside 9-(1,3-dihydroxy-2-propoxymethyl)guanine. Antimicrob Agents Chemother. 1983 May;23(5):676-82.

32. Wiltshire J. Hirankarn S, Farrell C, Paya C, Pescovitz MD, Humar A, Dominguez E, Washburn K, Blumberg E, Alexander B, Freeman R, Heaton N; Valganciclovir Solid Organ Transplant Study Group. Pharmacokinetic profile of ganciclovir after its oral administration and from its prodrug, valganciclovir, in solid organ transplant recipients. Clin Pharmacokinet. 2005;44(5):495-507.

33. Glesby MJ, Hoover DR, Weng S et al. Use of antiherpes drugs and the risk of Kaposi´s sarcoma: data from Multicenter AIDS Cohort Study. J Infect Dis 1996; 173: 1477-80.

34. Mocroft A, Youle M, Gazzard B, Morcinek J, Halai R and Phillips AN. Anti-herpesvirus treatment and risk of Kaposi´s sarcoma in HIV infection. AIDS 1996; 10: 1101-5.

35. Robles R, Lugo D, Gee L, Jacobson MA. Effect of antiviral drugs used to treat cytomegalovirus end-organ disease on subsequent course of previously diagnosed Kaposi's sarcoma in patients with AIDS. J Acquir Immune Defic Syndr Hum Retrovirol 1999; 20: 34-8.

36. Gantt S, Casper C. Human herpesvirus 8-associated neoplasms: the roles of viral replication and antiviral treatment. Curr Opin Infect Dis 2011; 24: 295-301.

37. Verucchi G, Calza L, Trevisani F, Zambruni A, Tadolini M, Giuliani R, Manfredi R, Andreone P, Chiodo F, Bernardi M. Human herpesvirus-8-related Kaposi's sarcoma after liver transplantation successfully treated with cidofovir and liposomal daunorubicin. Transpl Infect Dis 2005; 7: 34-7.

38. Wotherspoon AC, Doglioni C, Diss TC et al. Regression of primary low-grade B-cell gastric lymphoma of mucosa-associated lymphoid tissue after eradication of *Helicobacter pylori.* Lancet 1993; 342: 575-77.

39. Bayerdörffer E, Neubauer A, Rudolph B, Thiede Ch, Lehn N, Eidt S, Stolte M. Regression of primary gastric lymphoma of mucosa-associated lymphoid tissue after cure of *Helicobacter pylori* infection. Lancet 1995; 345:1591-94.

40. Raderer M, Pfeffel F, Pohl G, Mannhalter C, Vlencak J, Chott A. Regression of colonic low grade B cell lymphoma of the mucosa associated lymphoid tissue type after eradication of *Helicobacter pylori*. Gut 2000; 46: 133-5.

41. Huang GC, Sheu BS, Tsao CJ, Lin XZ, Su IJ. Eradication of *Helicobacter pylori* results in regression of B-cell low grade gastric MALToma with evident B-symptoms. Hepatogastroenterology 1998; 45: 2464-7

42. Makadzange AT, Ndhlovu CE, Takarinda K, Reid M, Kurangwa M, Gona P, Hakim JG. Early versus delayed initiation of antiretroviral therapy for concurrent HIV infection and cryptococcal meningitis in sub-saharan Africa. Clin Infect Dis 2010 Jun 1;50(11):153-8.

43. Ortega-Larrocea G, Espinosa E, Reyes-Terán G. Lower incidence and severity of cytomegalovirus-associated immune recovery uveitis in HIV-infected patients with delayed highly active antiretroviral therapy. AIDS. 2005 Apr 29;19(7):735-8.

44. Török ME, Yen NT, Chau TT, Mai NT, Phu NH, Mai PP, Dung NT, Chau NV, Bang ND, Tien NA, Minh NH, Hien NQ, Thai PV, Dong DT, Anh do TT, Thoa NT, Hai NN, Lan NN, Lan NT, Quy HT, Dung NH, Hien TT, Chinh NT, Simmons CP, de Jong M, Wolbers M. Farrar JJ. Timing of initiation of antiretroviral therapy in human immunodeficiency virus (HIV)--associated tuberculous meningitis. Clin Infect Dis. 2011 Jun;52(11):1374-83.

45. Abdool Karim SS, Naidoo K, Grobler A, Padayatchi N, Baxter C, Gray AL, Gengiah T, Gengiah S, Naidoo A, Jithoo N, Nair G, El-Sadr WM, Friedland G, Abdool Karim Q. Integration of antiretroviral therapy with tuberculosis treatment. N Engl J Med. 2011 Oct 20;365(16):1492-501. doi: 10.1056/NEJMoa1014181.

46. Martín-Carbonero L, Palacios R, Valencia E, Saballs P, Sirera G, Santos I, Baldobí F, Alegre M, Goyenechea A, Pedreira J, González del Castillo J, Martínez-Lacasa J, Ocampo A, Alsina M, Santos J, Podzamczer D, González-Lahoz J; Caelyx/Kaposi's Sarcoma Spanish Group Long-term prognosis of HIV-infected patients with Kaposi sarcoma treated with pegylated liposomal doxorubicin. Clin Infect Dis. 2008 Aug 1;47(3):410-7.

47. Volkow P, Lizano M, Carrillo-García A, Pérez-Montiel D, Garciadiego P. Triple secondary neoplasms: penis, lip and oral cavity in an AIDS patient treated withpegylated liposomal doxorubicin for cutaneous Kaposi's sarcoma. AIDS. 2014 Sep 24;28(15):2327-9.

V. CONTRIBUCION DEL PROYECTO EN EL AVANCE DEL CONOCIMIENTO EN SU PROPIA TEMATICA Y EN SU AREA DEL CONOCIMIENTO

En un estudio de mortalidad hospitalaria en pacientes con VIH en el Instituto Nacional de Cancerología durante 42 meses, el SK y el Linfoma No-Hodgkin ocuparon la primera causa de muerte en pacientes con VIH, cada una de estas patologías con 14 casos (36.8%). De los 14 pacientes que fallecieron con SK, en 13 de ellos fue secundario a compromiso pulmonar o enfermedad diseminada.

Hasta ahora no existen estudios clínicos prospectivos que demuestren el impacto clínico del uso de ganciclovir o valganciclovir (prodroga del ganciclovir en presentación oral) como parte del tratamiento de Sarcoma de Kaposi, en pacientes con enfermedad avanzada. Tampoco existen

estudios que establezcan si el tratamiento inicial del virus HVH-8 puede disminuir la respuesta inflamatoria asociada al SIRI, y por lo tanto tener un impacto en disminuir la mortalidad., Consideramos que el uso de un tratamiento antiviral contra VHH-8 podría abrir la puerta a nuevas opciones de tratamiento con el objetivo primario de disminuir la mortalidad en este grupo de pacientes.

VI. OBJETIVOS

Objetivo general:
Evaluar el papel del ganciclovir o valganciclovir en la fisiopatogenia de la infección por VIH en pacientes que cursen con SK y la respuesta clínica a éste
.

Objetivos particulares:

1. Objetivos Clínicos:
    a. Determinar si el manejo inicial con ganciclovir o la prodroga valganciclovir disminuye la incidencia de SIRI en pacientes con SIDA y SK.
    b. Determinar si el manejo con ganciclovir o la prodroga valganciclovir en pacientes con SIDA y SK disminuye la carga viral HVH-8
    c. Determinar si el manejo inicial con ganciclovir o la prodroga valganciclovir en pacientes con SIDA y SK pulmonar o diseminado grave yo linfadenopático disminuye la mortalidad atribuible.

2. Medir la respuesta inflamatoria en pacientes con SK avanzado y SIRI, con el uso de ganciclovir o valganciclovir y sin este a través de:

    a) Medir la concentración plasmática IL-6, IL-10, TNF-alfa, PCR y dímero D. Esta medición se realizara antes del tratamiento (basal) y en las semanas 1, 2, 4, 8, 12, 16, 24 y 48.
    b) Evaluarla frecuencia de linfocitos T CD4+, CD8+, células NK y monocitos pro-inflamatorios. Esta medición se realizara antes del tratamiento (basal) y en las semanas 1, 2, 4, 8, 12, 16, 24 y 48.

VII. HIPOTESIS O LINEAMIENTOS

La administración de ganciclovir y la supresión de la replicación de HVH-8 antes de la administración de TARAA, con o sin quimioterapia en pacientes con SIDA y SK diseminado, disminuirá la frecuencia de SIRI e impactara en disminuir la mortalidad atribuible a esta la enfermedad en pacientes.

VIII. METAS POR AÑO

Desarrollo de Protocolo:

"Supresión de la carga viral del HVH-8 antes del inicio del tratamiento antirretroviral altamente activo (TARRA) n pacientes con Sarcoma de Kaposi pulmonar y/o cutáneo diseminado grave y su impacto en el desarrollo de síndrome de recuperación inmune (SIRI) y en la mortalidad atribuible. Comparación con la terapia estar (TARAA) de inicio inmediato"
